# Endosome-associated Rab GTPases control distinct aspects of neural circuit assembly

**DOI:** 10.1101/2025.10.09.681358

**Published:** 2025-10-10

**Authors:** Katherine X. Dong, Hui Ji, David J. Luginbuhl, Liqun Luo, Colleen N. McLaughlin

**Affiliations:** Department of Biology, Howard Hughes Medical Institute, Stanford University, Stanford, CA 94305, USA

## Abstract

Neural circuit assembly relies on the precise regulation of cell-surface receptors that mediate signaling and adhesion. Endocytosis controls receptor activity and availability by internalizing and routing proteins through two main pathways: recycling back to the cell-surface or delivery to lysosomes for degradation. Rab GTPases direct receptors into these distinct pathways, but their specific contributions to circuit formation remain opaque. Using clonal analyses with null alleles, we dissected the roles of Rab-mediated trafficking to early, late, and recycling endosomes across multiple stages of circuit assembly *in vivo*. Our approach revealed that Rab5 and Rab11 regulated extensive and largely distinct developmental events, highlighting the pivotal roles of early endosome sorting and recycling pathways in circuit assembly. We found that as neurons mature, both the spatial distribution and abundance of specific endocytic compartments change to reflect evolving trafficking demands. Our findings underscore how distinct post-endocytic trafficking fates are necessary to build neural circuits.

## INTRODUCTION

Compartmentalization is a hallmark of eukaryotic cells and is essential for nearly all aspects of cellular physiology. Organelles partition cells into spatially distinct microdomains that concentrate factors to create biochemical environments necessary for cellular function. The endolysosomal system plays a vital role in subcellular organization. This dynamic network of membrane-bound vesicles integrates endocytic, biosynthetic, and degradative pathways to regulate metabolism, signaling, and protein trafficking (Settembre et al., 2013; Toshima & Toshima, 2024; Villaseñor et al., 2016; von Zastrow & Sorkin, 2021).

Neurons rely on the endolysosomal system to meet their extraordinary spatial and functional demands (Azarnia Tehran & Maritzen, 2022; Camblor-Perujo & Kononenko, 2022; Imoto & Watanabe, 2025; Yap et al., 2022). Endocytosis not only governs homeostatic processes like nutrient uptake but also underlies neuron-specific functions such as axon growth (Deshpande & Rodal, 2016; Lasiecka et al., 2014; Nishimura et al., 2003) and guidance (Pasterkamp & Burk, 2021; Zang et al., 2021), synaptic transmission (Kittler et al., 2005; Shioda et al., 2017), and neuronal polarity (Eichel et al., 2022). A major aspect of this regulation involves the selective internalization of cell surface receptors, which enables neurons to remodel their plasma membrane in response to internal and external cues. Following internalization, both soluble and membrane-anchored proteins transit to early endosomes. From there, proteins destined for degradation are trafficked through late endosomes to lysosomes (Cullen & Steinberg, 2018), while others are recycled to the plasma membrane or the Golgi apparatus (Toshima & Toshima, 2024; Grant & Donaldson, 2009). These two fates have diametrically opposed consequences: degradation causes signal termination, whereas recycling can enable signal reactivation. Thus, the endolysosomal system both shapes the neuronal surface proteome (McLaughlin et al., 2025) and plays a pivotal role in controlling signal transduction.

**Figure 1.**
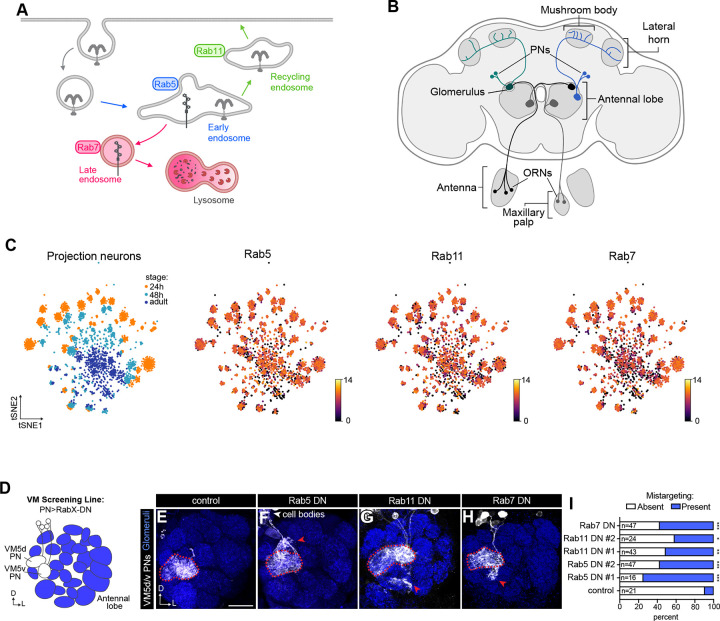
A subset of endosome-associated Rabs are expressed in olfactory projection neurons (PNs) and are involved in dendrite targeting. (A) Diagram of Rab GTPases and the endosomal compartments they predominantly localize to. (B) Schematic of the adult *Drosophila* brain, highlighting the olfactory system. ORNs, olfactory receptor neurons. PNs, projection neurons. Glomerulus on the far left depicts one-to-one matching between PNs and ORNs. (C) tSNE plots of developing PN single-cell RNA-seq (scRNA-seq) depicting stages profiled: 24h after puparium formation (APF), 48h APF, and adult (leftmost plot) and expression of endosome-associated Rab GTPases. Expression is in log_2_(CPM +1), where CPM stands for transcript counts per million reads. scRNA-seq data are from Xie et al., 2021. (D) Schematic of ventromedial (VM) screening line used in Rab GTPase dominant negative screen. GTP-binding defective dominant negative Rabs were expressed in all PNs using *VT033006-GAL4*, and targeting of VM5d/v was monitored with *GMR86C10-LexA>LexAop-mtdTomato*. (E–H) Representative images of indicated genotypes depicting phenotypes observed in dominant negative screen. Red dotted lines outline the VM5d/v glomeruli, and red arrows denote ectopic targeting. Scale bar, 20 μm. (I) Percent of antennal lobes with mistargeting in the Rab dominant negative screen. In this and all subsequent figures: D, dorsal; L, lateral. NCad (in blue) is used to label neuropil/glomeruli. * p < 0.05; ** p < 0.01; *** p < 0.001; n.s., not significant. n, the number of the antennal lobes quantified. Images of the antennal lobe were taken of 0–5 day-old adults unless otherwise noted.

Rab proteins comprise a highly conserved family of small GTPases that control intracellular protein trafficking (Wandinger-Ness & Zerial, 2014; Zerial & McBride, 2001). Rabs direct the post-endocytic itineraries of internalized cargos by compartmentalizing endocytic functions and coordinating transport to distinct endocytic vesicles such as early endosomes, recycling endosomes, late endosomes, and lysosomes (Bucci et al., 1992, 2000; Rink et al., 2005; Ullrich et al., 1996; Wandinger-Ness & Zerial, 2014; Zerial & McBride, 2001). Each Rab typically localizes to a specific membrane compartment (such as Rab5 to early endosomes; Figure 1A), where it functions as a molecular switch, cycling between an inactive, cytosolic GDP-bound state and an active, membrane-associated, GTP-bound form (Stenmark et al., 1994; Zerial & McBride, 2001). In their GTP-bound state, Rabs recruit effector proteins to coordinate trafficking events such as vesicle transport, tethering, and fusion (Jordens et al., 2005; Markgraf et al., 2007; Zerial & McBride, 2001). Thus, Rabs serve as essential sorting regulators that control the abundance, localization, and signaling activities of cell surface receptors. Although endocytic Rabs have been linked to neurodevelopmental disorders (Lamers et al., 2017; Meng et al., 2023) and processes (Saxena et al., 2005; Khodosh et al., 2006; Kawauchi et al., 2010; Rodal et al., 2011; Takano et al., 2012, 2014; Falk et al., 2014; K.-Y. Wu et al., 2014; Taylor et al., 2015; Liu et al., 2017; Furusawa et al., 2017), much prior work has examined the function of individual Rabs in isolated developmental events or in *in vitro* systems. Thus, we lack a comprehensive understanding of how distinct Rabs contribute to the assembly of intact neural circuits *in vivo*.

Here, we used *Drosophila* second-order olfactory projection neurons (PNs) to systematically evaluate the roles of Rab-mediated early, late, and recycling endocytic fates during circuit formation. PNs relay sensory information from the primary sensory neurons, olfactory receptor neurons (ORNs), to higher-order brain centers (Figure 1B). Assembly of the olfactory circuit begins with dendritic patterning of ~50 distinct PN types, which are largely derived from one of two main lineages: anterodorsal (adPNs) and lateral (lPNs) neuroblasts. Within each lineage, distinct types of PNs are generated in an invariant birth order and project their dendrites to stereotyped loci within the developing antennal lobe and their axons to higher-order brain centers (Jefferis et al., 2001, 2004; Lin et al., 2012; Marin et al., 2002; Yu et al., 2010). Subsequently, axons of each of the ~50 ORNs enter the antennal lobe and form one-to-one synaptic connections with PN dendrites at anatomically stereotyped positions, which will eventually become glomeruli (Vosshall & Stocker, 2007; Wilson, 2013).

This system enabled us to compare the functions of multiple Rabs within the same cell-types spanning multiple aspects of circuit assembly. We identified endocytic Rabs critical for circuit assembly using combined transcriptomic analysis and loss-of-function screening approach. Comprehensive clonal analysis of Rabs, each associated with mostly distinct endocytic compartments, revealed that each Rab, and its related endocytic fate, is critical for distinct aspects of circuit assembly and neuronal morphogenesis. These findings demonstrate that even within a single neuronal subtype, endocytic sorting pathways are differentially employed to control distinct aspects of connectivity.

## RESULTS

### Olfactory PNs express a subset of endosome-associated Rabs

The *Drosophila* genome encodes 33 Rab genes, 23 of which have direct human orthologs (Chan et al., 2011; Kohrs et al., 2021), offering reduced genetic redundancy compared to humans which encode 66 Rabs. We began by using our single-cell RNA-seq (scRNA-seq) data (Xie et al., 2021) to evaluate expression of all Rab GTPases in PNs corresponding to early (24h APF [after puparium formation]) and mid- (48h APF) development. We found that developing PNs express a subset of Rabs with some exhibiting dynamic expression across these two stages and others showing consistent expression (Figure 1-figure supplement 1A). Most endosome-associated Rabs had one of two expression patterns. Early endosome-associated (*Rab4, Rab5*) ( Gorvel et al., 199; van der Sluijs et al., 1992; Wandinger-Ness & Zerial, 2014), recycling endosome-associated (*Rab11*) (Ullrich et al., 1996; Zulkefli et al., 2019), and late endosome-associated (*Rab7*) (Rink et al., 2005) Rabs were ubiquitously and highly expressed in PNs (Figure 1C and Figure 1-figure supplement 1A), whereas early-endosomal *Rab21, Rab35* (Allaire et al., 2010; Kouranti et al., 2006; Simpson et al., 2004) displayed lower PN expression (Figure 1-figure supplement 1A). Finally, neither *Rab9* (late endosomes) (Lombardi et al., 1993) nor *Rab10* (recycling endosomes) (Chen et al., 2006; Etoh & Fukuda, 2019) were robustly expressed in PNs at any developmental stage (Figure 1-figure supplement 1A). Our analyses indicate that PNs express many, but not all, endosome-associated Rabs.

To begin evaluating the function of these GTPases, we performed a dominant negative (DN) screen of endosome-associated Rabs. We focused on those that are moderately to highly expressed in PNs and are associated with trafficking cargos through distinct endocytic compartments. Rab2, for instance, can associate with degradative compartments but was excluded from our screen due to its primary functions in axonal transport of lysosomes (Lund et al., 2021), delivery of lysosomal proteins to the lysosomal system (Lund et al., 2018), and autophagy (Ding et al., 2019; Zhou et al., 2022). We expressed GDP-locked versions of each Rab, which are designed to compete with the wild-type Rab for access to guanine nucleotide exchange factors (GEFs) or effectors, in all PNs and evaluated dendrite targeting to the VM5d and VM5v glomeruli (Figure 1D, E). Expressing DN versions of Rab4, Rab21, or Rab35 led to mild dendrite targeting defects typically with only a few branches extending beyond the VM5d/v glomerular boundary (Figure 1-figure supplement 1B–F). Expressing DN forms of Rab5, Rab11, or Rab7, on the other hand, resulted in stronger dendrite mistargeting (Figure 1E–I). Although impairing each of these Rabs caused PN dendrites to target to ectopic glomeruli neighboring VM5d/v, the spatial location of mistargeting varied. For instance, disrupting Rab5 function caused dendrites to spread in dorsal, lateral, and ventral directions (Figure 1F), whereas dendrites primarily mistargeted to ventral glomeruli when Rab11 or Rab7 were disrupted (Figure 1G, H).

Rab11 is associated with slow endosomal recycling (Takahashi et al., 2012; Ullrich et al., 1996; Zulkefli et al., 2019) from recycling endosomes to the plasma membrane whereas both Rab4 and Rab35 function in parallel fast recycling pathways directly from early endosomes to the cell surface (Allaire et al., 2010; Kouranti et al., 2006; van der Sluijs et al., 1992). The stronger phenotype observed upon Rab11 interference implies that the slow recycling route may be the predominant recycling pathway used in developing PNs. Further, among the early endosome-associated Rabs, Rab5 disruption produced stronger phenotypes than Rab21 (Figure 1F, I and Figure 1-figure supplement D, F). These findings are consistent with the idea that Rab5 regulates trafficking of a broad set of cargos, whereas Rab21 is thought to act more selectively on fewer cargos (Del Olmo et al., 2019; Pellinen et al., 2006; Shikanai et al., 2023). Together, these data indicate that the GTPase activity of a subset of endosome-associated Rabs is critical for PN dendrite targeting.

We wanted to leverage the mistargeting phenotypes observed upon disruption of Rab5, Rab7, and Rab11 to evaluate how distinct post-endocytic pathways contribute to circuit assembly. However, our initial analyses may have technical (e.g., late GAL4 driver expression) or biological (e.g., DN Rabs sequestering GEFs shared by multiple Rab proteins) limitations that could mask the full extent of Rab-specific contributions to this process. We circumvented this by performing clonal analyses in PNs homozygous for null alleles of *Rab5*, *Rab7*, or *Rab11*, which allowed us to dissect the individual roles of each Rab in PN development.

### Rab5 regulates multiple features of PN development

Early endosomes are the first major sorting station of internalized cargos. Rab5 controls early endosome biogenesis (Zeigerer et al., 2012), cargo trafficking (Bucci et al., 1992; Gorvel et al., 1991), and plays a key role in endosomal maturation toward late endosomes (Rink et al., 2005). As such, Rab5 has been implicated in dendrite arborization (Satoh et al., 2008) and targeting (Sakuma et al., 2014) in *Drosophila*, as well as in neuronal polarity (Guo et al., 2016) and dendrite development (Moya-Alvarado et al., 2018) in cultured mammalian neurons.

**Figure 2.**
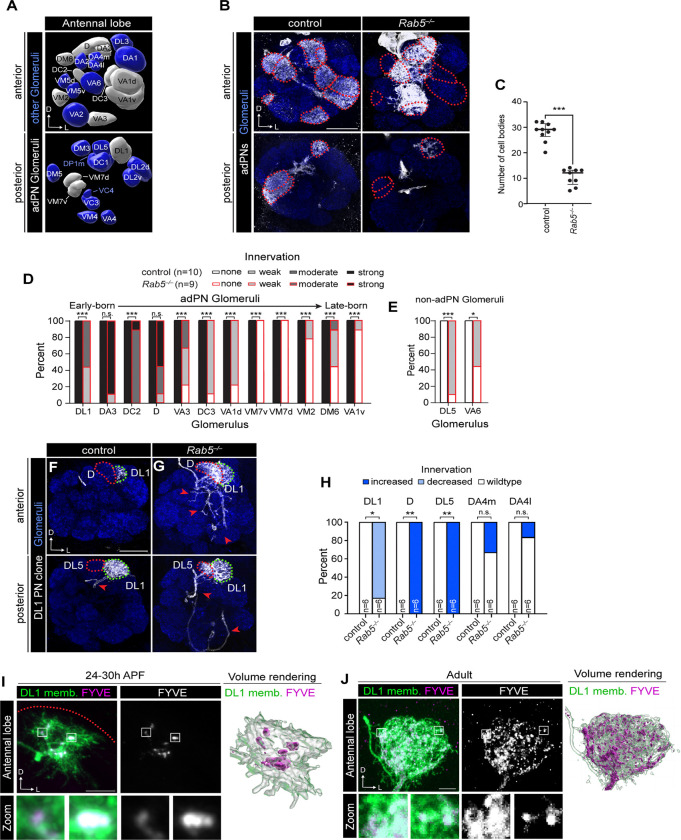
Rab5 regulates multiple features of PN dendrite development. (A) Volume rendering of a subset of glomeruli in the adult antennal lobe depicting those targeted by larval-born PNs from the anterodorsal lineage, hereafter adPNs, in grey, and other glomeruli in blue. (B) Representative images of dendrites from adPN neuroblast clones of indicated genotypes. Red dotted lines encircle adPN glomeruli. (C) Quantification of the number of cell bodies in adPN neuroblast clones in controls (n=10) and *Rab5*^−/−^ mutants (n=9). (D, E) Quantification of percent of antennal lobes with each category of dendrite innervation to adPN glomeruli (D) and glomeruli targeted by PNs other than larval-born adPNs (E). (F, G) Representative images of DL1-PN single-cell clone dendrites of indicated genotypes. Red dotted lines outline ectopically innervated glomeruli and green dotted lines outline DL1 glomerulus, and red arrows denote DL1-PN axon. (H) Quantification of percent of antennal lobes with altered dendrite innervation compared to controls. (I) Airyscan super resolution images depicting the localization of FYVE-mCherry expressed in a DL1-PN single-cell clone (left), single-channel FYVE image (middle), and 3D volume rendering (right) at 24–30h APF. (J) Representative images depicting the localization of the early endosome marker FYVE-mCherry expressed in a DL1-PN single-cell clone (left), single-channel FYVE image (middle), and 3D volume rendering (right) at the adult stage. Scale 20 μm (B, F); 5 μm (I, J)

Given the pleiotropic function of Rab GTPases, we sought to further define neurodevelopmental roles of *Rab5* by generating PN clones homozygous for a Rab5 null allele in single PNs or multiple PNs originating from the same lineage (neuroblast clones) using the mosaic analysis with repressible cell marker (MARCM) system (Lee & Luo, 1999; Wu & Luo, 2006b) (Figure 2-figure supplement 1A, B). We focused on the larval-born adPN neuroblast lineage (hereafter adPN clones) and the DL1-PN single-cell clones, whose development and targeting patterns are well characterized (Jefferis et al., 2001, 2004) (Figure 2A). Loss of Rab5 in adPN neuroblast clones caused a significant decrease in the number of labeled cells (Figure 2B, C), indicating that *Rab5* mutants are undergoing cell death or may have defective cell proliferation. Since PN dendrite targeting and birth order are stereotyped (Jefferis et al., 2001; Yu et al., 2010), we differentiated between these two possibilities by evaluating which glomeruli lose innervation in *Rab5* adPN clones. If PNs are dying, we would expect to observe a loss of innervation across all glomeruli, whereas proliferation defects would disproportionately affect later-born PNs. In line with the latter explanation, we observed a loss of innervation to glomeruli normally targeted by later-born adPNs compared to those that are targeted by earlier-born adPNs (Figure 2D). Beyond loss of innervation, *Rab5* mutants exhibited ectopic innervation of glomeruli not normally targeted by adPN dendrites (Figure 2B, E and Figure 2-figure supplement 1C). These data indicate that Rab5 is critical for both neuronal proliferation and dendrite targeting.

We next tested the cell-autonomous role for *Rab5* in PN dendrite targeting. We generated single-cell DL1-PN clones and quantified dendrite targeting to this glomerulus. Loss of Rab5 in DL1-PN clones resulted in ectopic innervation into the neighboring glomeruli D and DL5 as well as reduced innervation to the DL1 glomerulus (Figure 2F–H), consistent with a previous study (Sakuma et al., 2014). Although the deficits did not reach statistical significance, we also observed consistent mistargeting to DA4m and DA4l glomeruli (Figure 2H) in *Rab5* single-cell clones. Thus, Rab5 cell-autonomously regulates PN dendrite targeting.

Finally, we wanted to visualize the distribution of early endosomes in PN dendrites. Using MARCM, we expressed mCherry-2xFYVE, which predominantly accumulates on early endosomes (Gaullier et al., 1998; Gillooly et al., 2000), only in DL1-PNs at an early developmental timepoint and in adults. During development, there were relatively few early endosomes in PN dendrites, and they predominantly localized to more central dendrite regions and not the growing distal processes (Figure 2I). Adult dendrites, on the other hand, were filled with many small early endosomes and larger clusters (Figure 2J). These findings indicate that the early endosome population expands in tandem with dendritic arbor growth.

### Rab5 is required for axon guidance and terminal development

**Figure 3.**
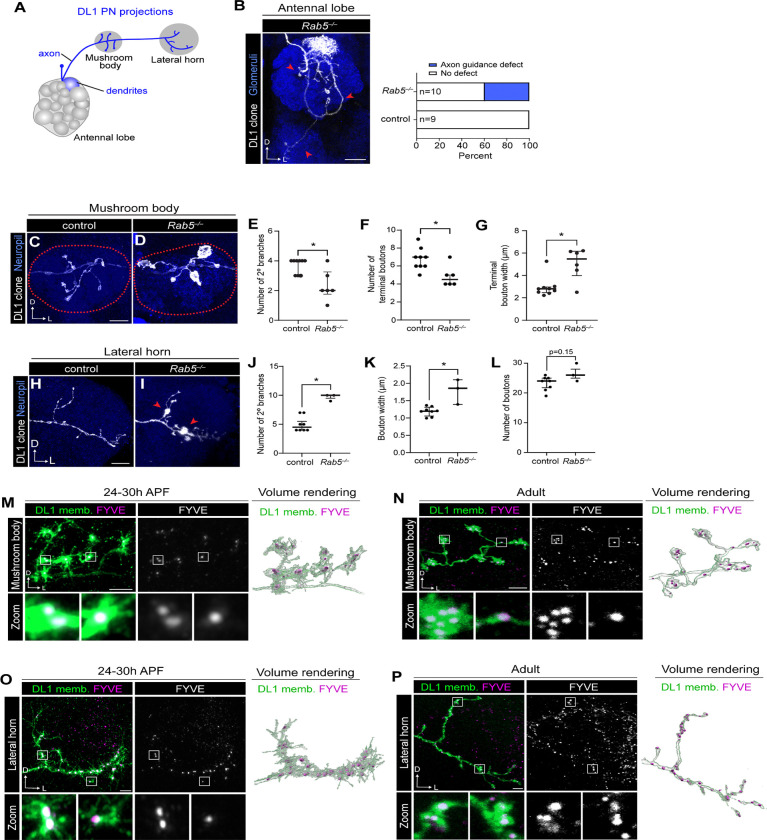
Rab5 is required for axon guidance and terminal maturation. (A) Schematic depicting DL1-PN dendrites innervating the DL1 glomerulus and axon projecting to the mushroom body and lateral horn. (B) Representative image and quantification of the DL1-PN axon guidance defect where the axon does not project to the mushroom body or lateral horn (left) and quantification of the number of antennal lobes where this phenotype was observed (right). Red arrows denote DL1-PN axons terminating in ectopic neuropil regions. (C, D) Representative image of control (C) or *Rab5*^−/−^ mutant (D) DL1-PN axons at the mushroom body. Dotted lines denote the border of the mushroom body. (E–G) Quantification of number of secondary branches (E), number of terminal boutons (F), and average terminal bouton width (G) in each DL1-PN mushroom body axon for controls (n=9) and *Rab5*^−/−^ mutants (n=6). (H, I) Representative image of a control (H) or *Rab5*^−/−^ mutant (I) DL1-PN axon at the lateral horn. Red arrows denote enlarged boutons. (J–L) Quantification of number of secondary branches (J), average bouton width (K), and number of boutons (L) in each DL1-PN axon at the lateral horn for controls (n=8) and *Rab5*^−/−^ mutants (n=3). (M) Airyscan super resolution images depicting the mushroom body localization of the early endosome marker FYVE-mCherry expressed in a DL1-PN single-cell clone (left), single-channel FYVE image (middle), and 3D volume rendering (right) at 24–30h APF. (N) Representative images depicting the mushroom body localization of FYVE-mCherry expressed in a DL1-PN single-cell clone (left), single-channel FYVE image (middle), and 3D volume rendering (right) at the adult stage. (O) Airyscan super resolution images depicting the lateral horn localization of FYVE-mCherry expressed in a DL1-PN single-cell clone (left), single-channel FYVE image (middle), and 3D volume rendering (right) at 24–30h APF. (P) Representative images depicting the lateral horn localization of FYVE-mCherry expressed in a DL1-PN single-cell clone (left), single-channel FYVE image (middle), and 3D volume rendering (right) at the adult stage. Note that puncta outside of lateral horn axons are in other nearby cell types. Scale bar, 20 μm (B); 10 μm (C, H); 5 μm (M–P)

PN axons exit the antennal lobe and project to the mushroom body and lateral horn (Figure 3A) where they elaborate branches and form synaptic boutons. Rab5 has been implicated in regulating axon elongation (Falk et al., 2014; Sakuma et al., 2014), fasciculation (K.-Y. Wu et al., 2014) and guidance (Sakuma et al., 2014; K.-Y. Wu et al., 2014); however, its roles in other developmental processes are largely undefined. Our ability to generate DL1-PN mutant clones enabled us to evaluate the function of Rab5 in axon growth and guidance as well as finer-scale morphological processes. Consistent with previous work (Sakuma et al., 2014), 40% of the DL1-PN clones terminated in the vicinity of the antennal lobe rather than projecting to higher-order regions (Figure 3B), indicating that Rab5 is important for axon guidance.

We next assayed the morphology of the *Rab5* axons that made it to higher-order brain centers. Though the precise location of axon branches within the mushroom body is stochastic, these axons do have stereotyped bouton number and morphology (Figure 3C). Compared to controls, *Rab5* mutant DL1-PN axons had fewer secondary branches (Figure 3E) as well as a reduction in the number of terminal boutons (Figure 3F). Yet, the terminal boutons in *Rab5* mutants were nearly 2-fold larger than those of controls (Figure 3G), suggesting Rab5 regulates the trafficking of cell-surface proteins that normally restrain bouton growth. These results indicate that *Rab5* is required for axons to elaborate the correct number and size of boutons and branches.

DL1-PN projections to the lateral horn are stereotyped and exhibit a characteristic “L-shaped” branching pattern, composed of a shorter dorsal and longer lateral branch (Figure 3H) each of which contains relatively few higher-order branches (Figure 3H, J). In contrast to the mushroom body, *Rab5* mutant lateral horn projections formed more secondary branches than controls (Figure 3I, J). Additionally, boutons in *Rab5* mutants were significantly larger but not more numerous (Figure 3K, L). These data indicate that Rab5 restrains multiple features of axon growth and terminal morphology in the lateral horn. Taken together, the distinct branching phenotypes observed in the mushroom body versus lateral horn suggest that Rab5 may regulate the trafficking of a distinct set of cargos in each axonal compartment.

To understand where Rab5 could be acting to carry out its axonal functions, we mapped the distribution of early endosomes in each axon projection. Early endosomes had similar distributions in both developing and adult mushroom body projections, accumulating in terminal boutons (Figure 3 M, N). Similar to their distribution in dendrites, early endosomes in the lateral horn were distributed throughout the axon but were not present in actively growing processes (Figure 3O). Early endosomes were likewise observed throughout the adult lateral horn terminal, with numerous puncta present both within boutons and the inter-bouton regions (Figure 3P). These data indicate that early endosome distribution stays relatively constant in developing and adult PN axons.

### Rab7 plays a minor role in PN dendrite targeting

Following entry into early endosomes, internalized cargo is directed toward one of two main destinations: lysosomes or recycling endosomes. However, the relative contributions of these distinct trafficking outcomes to the development of the same neuron *in vivo* remain largely opaque. To address this, we began by assaying Rab7 function in PN development. Rab7 predominantly localizes to late endosomes where it facilitates conversion of early endosomes to late endosomes (Rink et al., 2005), regulates late endosome fusion with lysosomes (Bucci et al., 2000; Vanlandingham & Ceresa, 2009; Xing et al., 2021), and promotes autophagosome maturation (Hyttinen et al., 2013).

The *Rab7* null mutant is a GAL4 knock-in into the endogenous *Rab7* locus (Chan et al., 2011) and is, therefore, incompatible with traditional MARCM approaches as they rely on the GAL4/UAS system to label mutant cells (Lee & Luo, 1999; Wu & Luo, 2006b). To circumvent this and analyze Rab7 function in PN development, we performed clonal analyses with a second binary expression system, the Q system (Potter et al., 2010; Potter & Luo, 2011), referred to as QMARCM (Potter et al., 2010). QMARCM is comparable to GH146-GAL4-based MARCM (Potter et al., 2010) but uses GH146-QF to drive QUAS transgenes carrying membrane markers or rescue transgenes.

We first quantified the number of adPN cell bodies and found that *Rab7* is dispensable for neurogenesis and/or neuronal survival (Figure 4-figure supplement 1A). Further, loss of *Rab7* resulted in mild dendrite targeting defects in neuroblast clones (Figure 4-figure supplement 1B, C, E, F). A few glomeruli (DL1, DC2, and DM6) had a reduction in innervation compared to controls (Figure 4-figure supplement 1C, E), but the rest were unchanged. To verify that Rab7 was responsible for these phenotypes, we generated a *QUAS-mCherry-Rab7* transgene and re-expressed it in adPN clones. Expression of mCherry-Rab7 in *Rab7* adPNs rescued the innervation defects to the DL1 and DM6 glomeruli and partially rescued targeting to DC2 (Figure 4-figure supplement 1D, E). In addition to the decrease in innervation, *Rab7* mutants exhibited minor mistargeting to non-adPN glomeruli (Figure 4-figure supplement 1F). While some of the mistargeting phenotypes could be rescued by re-expression of *mCherry-Rab7* others could not (Figure 4-figure supplement 1D, F), indicating that either precise Rab7 levels are important for dendrite targeting or that *Rab7* loss in non-ad PNs could exert non-autonomous effects on neighboring neurons. This data suggests that Rab7 regulates dendrite targeting of a subset of PNs but is not broadly required for this process.

**Figure 4.**
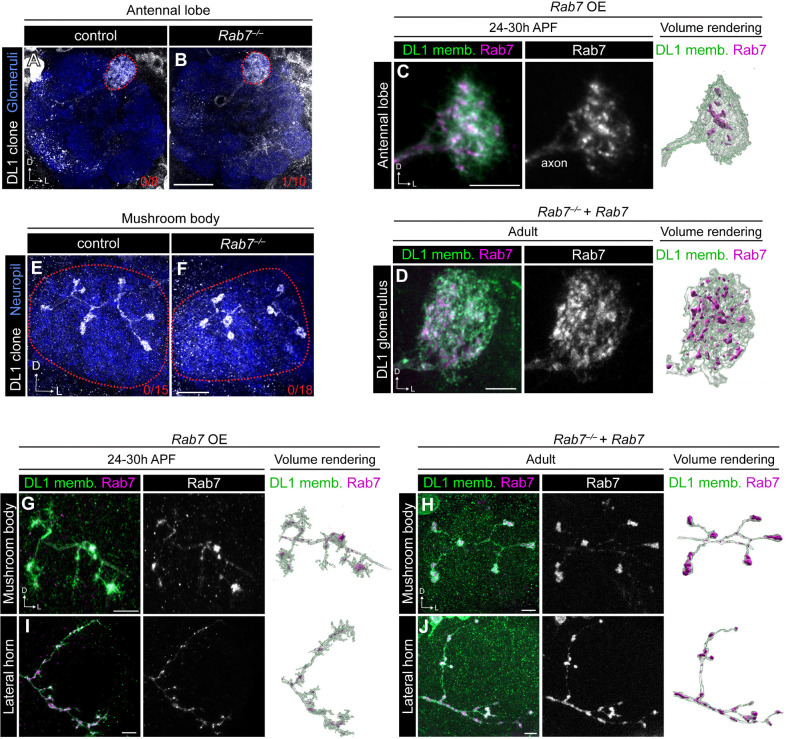
Rab7 plays a minor role in PN development. (A, B) Representative images of dendrite targeting of DL1-PN single-cell clones in control (A) and *Rab7*^−/−^ mutant (B) antennal lobes. Red numbers in the right corner of images denote DL1-PN dendrite mistargeting phenotypic penetrance. (C) Airyscan super-resolution image depicting localization of mCherry-Rab7 in developing DL1-PN dendrites (left), single-channel image of Rab7 (middle), and 3D volume rendering of Rab7 distribution (right). (D) Representative image of localization of mCherry-Rab7 in a *Rab7*^−/−^ mutant background in adult DL1-PN single-cell dendrites (left), single-channel image of Rab7 (middle), and 3D volume rendering of Rab7 distribution (right). (E, F) Representative images of mushroom body projections of control (E) and *Rab7*^−/−^ mutant (F) DL1-PN single-cell clones. Red numbers in the right corner of images denote DL1-PN axon morphogenesis phenotypic penetrance. (G) Airyscan super-resolution image depicting localization of mCherry-Rab7 in a developing DL1-PN axon at the mushroom body (left), single-channel image of Rab7 (middle), and 3D volumes rendering of Rab7 distribution (right). (H) Representative image of localization of mCherry-Rab7 in a *Rab7*^−/−^ mutant background in an adult DL1-PN axon at the mushroom body (left), single-channel image of Rab7 (middle), and 3D volume rendering of Rab7 distribution (right). (I) Airyscan super-resolution image depicting localization of mCherry-Rab7 in a developing DL1-PN axon at the lateral horn (left), single-channel image of Rab7 (middle), and 3D volume rendering of Rab7 distribution (right). (J) Representative image of localization of mCherry-Rab7 in a *Rab7*^−/−^ mutant background in an adult DL1-PN axon at the lateral horn (left), single-channel image of Rab7 (middle), and 3D volume rendering of Rab7 distribution (right). Scale bar, 20 μm (B); 10 μm (F) 5 μm (C, D, G-J)

Does Rab7 have cell-autonomous roles in dendrite targeting? While neuroblast clones showed reduced innervation to the DL1 glomerulus, *Rab7* mutant DL1-PN single-cell clones did not exhibit defects in dendrite targeting (Figure 4A, B). The failure to recapitulate the loss of innervation observed in neuroblast clones could indicate that (1) Rab7 is non-autonomously required for this process or (2) Rab7 perdurance may occur in the DL1-PN single-cell clones. Our initial findings that expressing Rab7-DN in all PNs caused dendrite targeting defects (Figure 1H) imply that Rab7 may be non-autonomously required for dendrite targeting. To confirm this and circumvent potential RNA/protein perdurance in DL1-PN MARCM clones, we used a DL1-PN GAL4 driver to express Rab7-DN which validated that Rab7 is not required in DL1 PNs to regulate their dendrite targeting (Figure 4-figure supplement 2A, B). Thus, the DL1-PN dendrite phenotypes in adPN neuroblast clones are likely caused by disrupting Rab7 in other PNs, suggesting that Rab7 is non-autonomously required for dendrite targeting of some PNs such as DL1-PNs.

Finally, we examined Rab7 localization in developing and adult dendrites. To accomplish this, we re-expressed the mCherry-Rab7 transgene in *Rab7* mutant DL1-PN dendrites and evaluated mCherry-Rab7 localization. In adult dendrites, we found that Rab7 is punctate and present throughout the dendritic arbor (Figure 4D); however, we were unable to obtain reliable membrane GFP expression at 24–30h APF using the QMARCM system. We bypassed this limitation by generating and expressing a UAS version of mCherry-Rab7 in DL1-PNs using MARCM. During development, Rab7 was similarly present throughout the dendritic arbor (Figure 4C), suggesting that developing and adult dendrites may have comparable degradative needs.

### Rab7 is not required for PN axon development

Does Rab7 play a role in axon development? To test this, we evaluated DL1-PN axon morphology in the mushroom body and lateral horn and found that *Rab7* mutant DL1-PN axons did not differ in morphology or branching compared to control DL1-PN axons in the mushroom body (Figure 4E, F) or lateral horn (Figure 4-figure supplement 1G). Consistently, expression of Rab7-DN in DL1-PNs did not alter any aspect of axonal morphology (Figure 4-figure supplement 2C–F). Thus, Rab7 is dispensable for DL1-PN axon development.

Using the same genetic strategies as in dendrites, we evaluated Rab7 localization in mushroom body and lateral horn axons. In both developing and adult mushroom body axons, Rab7 appeared to be in large clusters (Figure 4G, H) within the boutons. Rab7 was abundant in both developing and adult lateral horn projections (Figure 4I, J), with large clusters observed in adult boutons—similar to the pattern seen in the mushroom body (Figure 4J). Overall, while Rab7 is expressed in developing neurons, Rab7-mediated degradation does not have a major role in regulating axon or dendrite development.

### Rab11 has PN type-specific roles in dendrite targeting and innervation

Rab11 predominantly localizes to recycling endosomes and facilitates cargo recycling to the plasma membrane via this vesicular pool (Zulkefli et al., 2019). Thus, to understand how Rab11-mediated endosomal recycling contributes to circuit assembly, we began by testing Rab11’s role in PN dendrite targeting.

**Figure 5.**
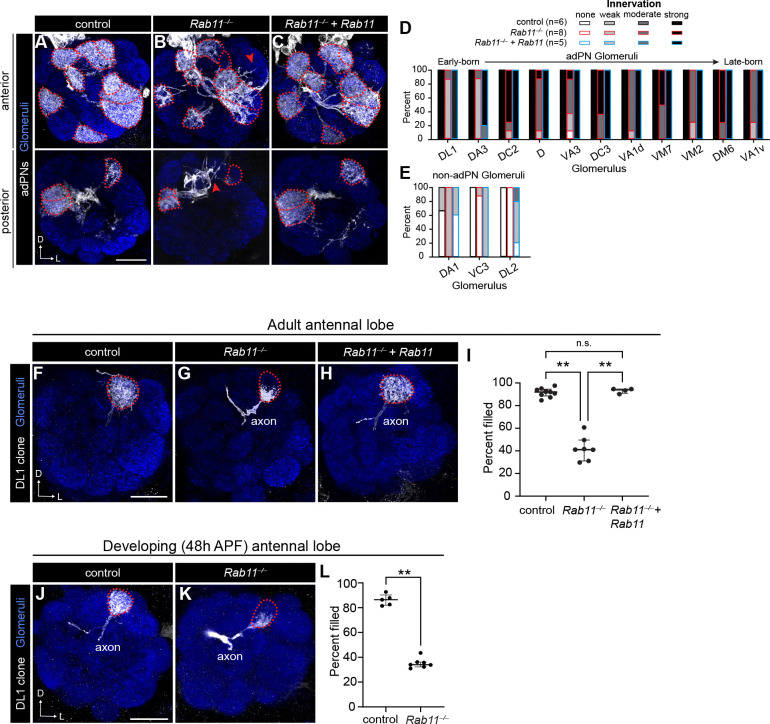
Rab11 has cell-type-specific roles in PN dendrite targeting and innervation. (A–C) Representative images of dendrite targeting of adPN neuroblast clones in control (A), *Rab11*^−/−^ mutant (B), and *Rab11* rescue (C) antennal lobes. (D, E) Quantification of percent of antennal lobes with each category of dendrite innervation to adPN glomeruli (D) and glomeruli targeted by PNs other than larval-born adPNs (E). (F–H) Representative images of control (F), *Rab11*^−/−^ mutant (G), and *Rab11* rescue (H) DL1-PN single-cell clone dendrites. (I) Percent of the DL1 glomerulus filled by DL1-PN dendrites of controls (n=9), *Rab11*^−/−^ mutants (n=7), and *Rab11* rescues (n=4). (J, K) Representative images of control (J) and *Rab11*^−/−^ mutant (K) DL1-PN single-cell clone dendrites at 42–48h APF. (L) Percent of the DL1 glomerulus filled by DL1-PN dendrites of controls (n=5) and *Rab11*^−/−^ mutants (n=7) at 42–48h APF. Scale bar, 20 μm (A, F); 15 μm (J)

*Rab11* mutant adPNs exhibited decreased dendrite innervation into several adPN glomeruli as well as ectopic innervation into other non-adPN glomeruli (Figure 5A, B, D, E and Figure 5-figure supplement 1A), indicating that Rab11 regulates PN dendrite targeting. Loss of innervation to adPN glomeruli was not caused by impaired proliferation or increased cell death, as there was no difference in the number of adPNs in *Rab11* mutants compared to controls (Figure 5-figure supplement 1B). Re-expressing an mCherry-tagged Rab11 (Chen & He, 2022) in adPNs rescued the majority of the innervation defects (Figure 5C–E and Figure 5-figure supplement 1A), indicating that these defects are specific to *Rab11* loss. However, re-expression of Rab11 in *Rab11* adPN clones did cause some mistargeting to non-adPN glomeruli (Figure 5E and Figure 5-figure supplement 1A), suggesting that specific Rab11 levels may be important for the development of some PN types.

The loss of innervation we observed in neuroblast clones could reflect either dendrite mistargeting to incorrect glomeruli or a failure of these dendrites to fully innervate within their appropriate target. To distinguish between these possibilities, we evaluated DL1-PN single-cell clones. DL1-PN clones did not exhibit dendrite mistargeting (Figure 5F, G, I). Rather, their dendrites failed to fully innervate the DL1 glomerulus and instead were concentrated at its ventromedial region, a phenotype that was fully rescued by cell-autonomous re-expression of *Rab11* (Figure 5H, I). Since defects in endosomal recycling are associated with neurodegeneration (Kiral et al., 2018), we probed if this innervation defect was the result of a failure to elaborate during development or retraction/degeneration in the adult stage. To answer this, we characterized DL1-PN dendrites during development. Similar to the adult phenotype, dendrites failed to fill the DL1 glomerulus, with only ~30% coverage (Figure 5J–L). Together, these data indicate that Rab11 is required for dendrite elaboration.

Since *Rab11* mutant adPN neuroblast clones also exhibited ectopic targeting, we hypothesized that Rab11 could have cell-type-specific roles in dendrite targeting. Thus, we evaluated dendrite targeting in *Rab11* mutant DA1 or VA1d/DC3 neuroblast clones using *MZ19-GAL4-*based MARCM analysis. In neuroblast clones that contain labeled DA1-PNs, *Rab11* mutants exhibited reduced innervation into the DA1 glomerulus, along with ectopic targeting to mainly ventrolateral glomeruli (Figure 5-figure supplement 1C–E). Similarly, we observed a decrease in innervation into the VA1d and DC3 glomeruli and mistargeting to medial glomeruli in *Rab11* mutant neuroblast clones that contain labeled VA1d/DC3-PNs (Figure 5-figure supplement 1C–E). Importantly, *Rab11* mutants had a similar number of labeled cells as controls for both sets of clones (Figure 5-figure supplement 1F, G). Collectively, these data reveal that Rab11 has cell-type specific roles in PN dendrite targeting and innervation and suggest that Rab11 may regulate recycling of distinct cargos in each cell-type.

### Rab11 promotes axon maturation

**Figure 6.**
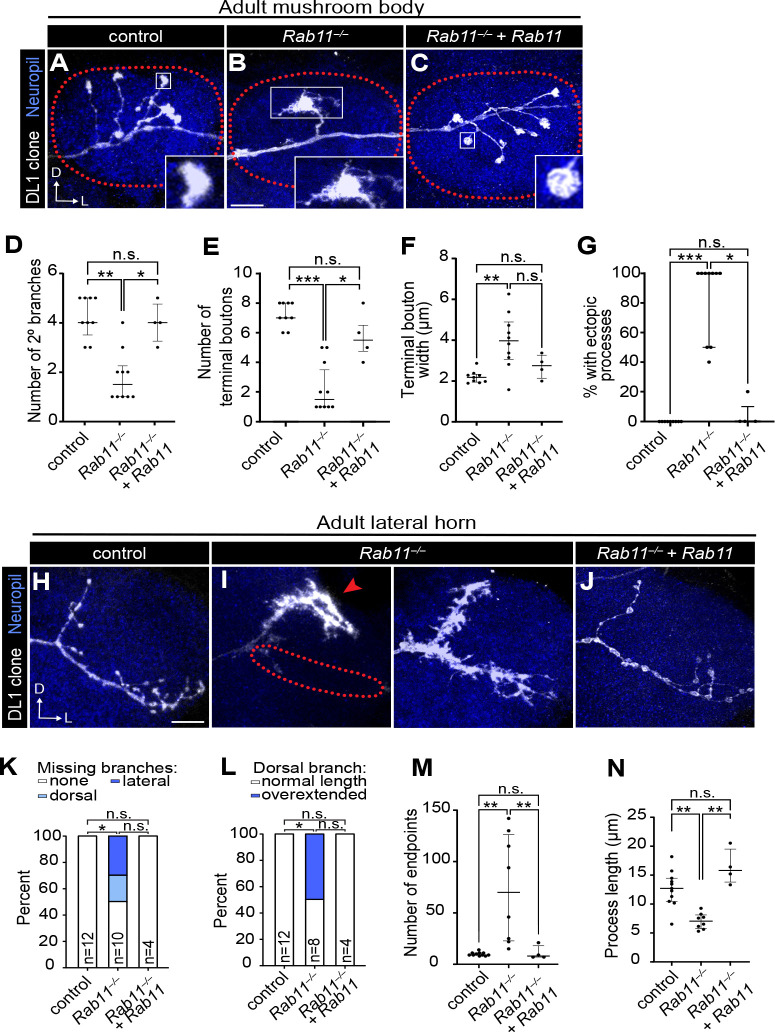
Rab11 promotes PN axon development. (A–C) Representative images of control (A), *Rab11*^−/−^ mutant (B), and *Rab11* rescue (C) DL1-PN axons at the mushroom body. Dotted lines denote the border of the mushroom body. (D–G) Quantification of the number of secondary branches (D), number of terminal boutons (E), average terminal bouton width (F), and percent of terminal boutons with ectopic processes (G) in each DL1-PN axon for controls (n=9), *Rab11*^−/−^ mutants (n=10), and *Rab11* rescues (n=4). (H–J) Representative images of control (H), *Rab11*^−/−^ mutant (I), and *Rab11* rescue (J) DL1-PN axons at the lateral horn. (K, L) Quantification of the percent of axons missing a main branch (K) or percent with an overextended dorsal branch (L). (M, N) Quantification of number of endpoints (M) or average process length (excluding main branches) (N) of each DL1-PN axon in the lateral horn of controls (n=11), *Rab11*^−/−^ mutants (n=8), and *Rab11* rescues (n=4). Some measures did not reach statistical significance due to a low sample number inherent with MARCM analysis. Scale bar, 10 μm (B, H)

Next, we probed the role of Rab11 in DL1-PN axons. In the mushroom body, *Rab11* mutant DL1-PN axons had fewer secondary branches and terminal boutons along with increased terminal bouton size (Figure 6A, B, D–F), similar to *Rab5* mutants (Figure 3E, F). However, *Rab11* mutant terminal boutons also exhibited thin ectopic processes emanating from them (Figure 6B, G), reminiscent of filopodia that are present during axon development (Jefferis et al., 2004; Zhu & Luo, 2004). Most loss-of-function phenotypes could be rescued by re-expressing *Rab11* in DL1-PNs, indicating that Rab11 is cell-autonomously required to promote mushroom body axon branching and restrain bouton size (Figure 6C, D–F).

Given the filopodia-like processes present in *Rab11* mutant axons, we asked whether this GTPase promotes axon maturation. DL1-PNs extend nascent axonal branches into the mushroom body around 12h APF which mature into defined collateral branches containing multiple boutons by approximately 50h APF(Jefferis et al., 2004; Zhu & Luo, 2004). To evaluate if *Rab11* mutant axon phenotypes are the result of maturation defects, we examined these axons at two developmental timepoints: 24–30h APF and 42–48h APF. At the earlier timepoint, control axons were collateralized and contained nascent boutons with several filopodia-like processes (Figure 6-figure supplement 1A). However, *Rab11* mutant axons had fewer secondary branches and, therefore, a reduced number of terminal boutons (Figure 6-figure supplement 1B, E), indicating that during development they fail to elaborate the correct number of branches. By 42–48h APF, control axons had fewer secondary branches and filopodia-like structures and were beginning to resemble the mature terminal (Figure 6-figure supplement 1C). By this later timepoint, *Rab11* mutants had lost most of their secondary branches and often contained a single branch or large bouton, similar to phenotypes observed in the adult stage (Figure 6B and Figure 6-figure supplement 1D) indicating that Rab11 is required for both axon branch formation and stability. Together, Rab11 is required for mushroom body axon maturation.

We next evaluated Rab11 function in lateral horn axons. Loss of *Rab11* resulted in multiple morphological defects in these projections. ~50% of *Rab11* mutant axons lacked either the dorsal or lateral branch (Figure 6H, I, left, K). Among those with a dorsal collateral, ~50% exhibited overextension of this process and some had a second dorsal branch (Figure 6I, left, L), which is occasionally observed in pupal stages but does not persist in adults (Jefferis et al., 2004). In addition to branching defects, *Rab11* mutant axons lacked boutons and were instead covered in many short filopodia-like processes (Figure 6I) similar to the nascent processes we observed in the mushroom body. We quantified this by measuring the number of endpoints and average length of each process emanating from the dorsal or lateral branches and found that *Rab11* mutant axons contained a significantly higher number endpoints and shorter process lengths than controls (Figure 6M, N). Many of these phenotypes were rescued by re-expressing *Rab11* in DL1 PNs (Figure 6J, K–N).

Next, we interrogated the origins of the *Rab11* mutant phenotypes by examining developing lateral horn axons to determine: (1) if the branching defects resulted from a failure to form branches or from retraction; and (2) whether the persistence of filopodia-like processes was due to delayed development. The lateral branch extends first (at ~18h APF) followed by the appearance of the dorsal collateral between 24–30h APF (Jefferis et al., 2004). By 42h APF, a more mature axonal morphology emerges, characterized by the loss of many filopodia-like projections and the appearance of rounded boutons (Figure 6-figure supplement 1G, I). Developing *Rab11* mutant axons never lost these nascent projections and also lacked either a dorsal or lateral branch (Figure 6-figure supplement 1H [right]), similar to the phenotypes at the adult stage. The proportion of *Rab11* mutants missing the lateral branch at the adult stage was approximately two and a half-fold higher than at either developmental stage, whereas the proportion of mutants missing the dorsal branch remained similar across all stages (Figure 6K, Figure 6-figure supplement 1K). These findings suggest that Rab11 plays distinct roles in lateral horn axon development by promoting dorsal branch formation and ensuring lateral branch stabilization. Moreover, the persistence of the filopodia-like processes in *Rab11* further supports the idea that this GTPase is essential for axon maturation. Collectively, our data demonstrates that Rab11-mediated recycling is required for axon maturation and morphological stability.

### Developmental dynamics of Rab11 distribution in PNs

**Figure 7.**
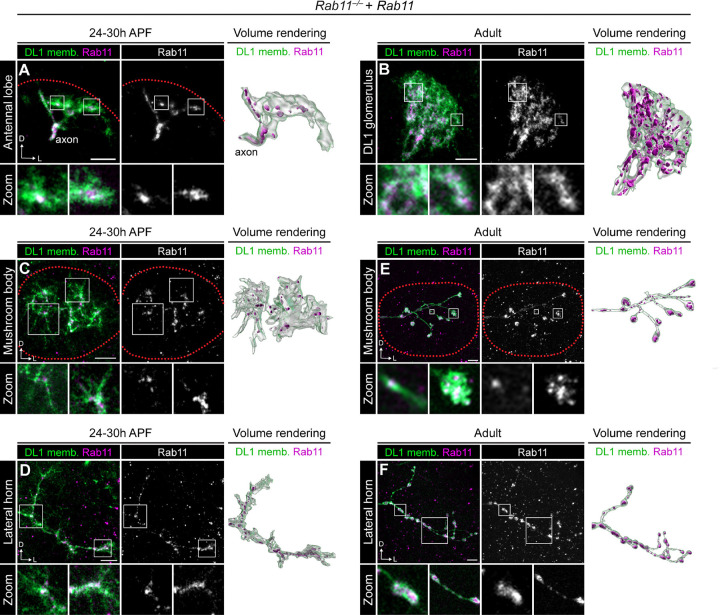
Developmental dynamics of Rab11 distribution in PNs. (A) Airyscan super resolution images depicting the localization of mCherry-Rab11 in a *Rab11*^−/−^ mutant background in a DL1-PN single-cell clone dendrites (left), single-channel Rab11 image (middle), and 3D volume rendering (right) at 24–30h APF. Zoom panels are single optical sections of areas indicated by white boxes. (B) Representative images depicting mCherry-Rab11 localization in a *Rab11*^−/−^ mutant background in a DL1-PN single-cell clone dendrites (left), single-channel Rab11 image (middle), and 3D volume rendering (right) at the adult stage. Zoom panels are single optical sections of areas indicated by white boxes. (C, D) Airyscan super resolution images depicting mCherry-Rab11 localization at the mushroom body (C) and lateral horn (D) in a *Rab11*^−/−^ mutant background in a DL1-PN single-cell clone (left), single-channel Rab11 image (middle), and 3D volume rendering (right) at 24–30h APF. Zoom panels are single optical sections of areas indicated by white boxes. (E, F) Representative images depicting mCherry-Rab11 localization at the mushroom body (E) and lateral horn (F) in a *Rab11*^−/−^ mutant background in a DL1-PN single cell clone (left), single-channel Rab11 image (middle), and 3D volume rendering (right) in the adult stage. Zoom panels are single optical sections of areas indicated by white boxes. Scale bar, 5 μm (A-F)

Since Rab11 was broadly required for PN development, we sought to visualize its distribution to gain insight into where it might be acting. Because re-expression of mCherry-Rab11 rescued most *Rab11* mutant phenotypes, we leveraged this genetic background to visualize Rab11 localization in developing and adult DL1-PN clones. During development, Rab11 was largely excluded from the distal dendritic processes and was instead concentrated at branch points, suggesting that recycling endosomes are enriched at central dendritic hubs during arborization (Figure 7A). In adult neurons, Rab11 was distributed throughout the arbor, including in distal processes (Figure 7B). The increased number and density of Rab11-positive compartments in adult dendrites suggests that as dendrites grow and elaborate there is a concomitant expansion of the recycling endosome network to support their functional demands.

We next examined Rab11 localization in axons. Similar to developing dendrites, Rab11 was enriched at axonal branchpoints in both mushroom body and lateral horn axons, with reduced localization to actively growing processes (Figure 7C, D). By the adult stage, Rab11 accumulated in boutons with fewer smaller puncta in the inter-bouton regions (Figure 7E, F). Rab11 enrichment in mature boutons of both lateral horn and mushroom body axons could reflect its well-defined role in synaptic vesicle recycling (Ivanova & Cousin, 2022). Altogether, these observations indicate that Rab11 localization is dynamically regulated across development likely reflecting changes in recycling needs or cargo distribution across time.

## DISCUSSION

**Figure 8.**
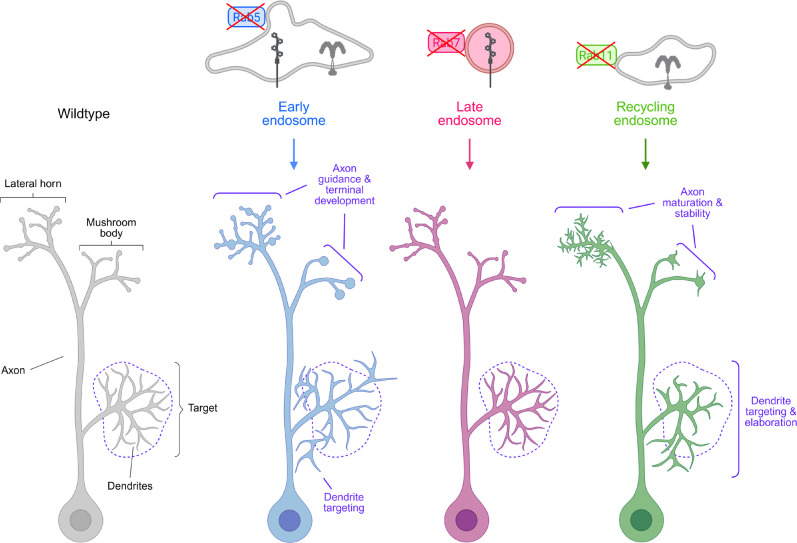
Endosome-associated Rab GTPases regulate distinct aspects of neuronal morphogenesis and circuit assembly. Summary model of the cell-autonomous functions of endosome-associated Rab GTPases in PN development.

Building functional neural circuits depends on the coordination of diverse cellular processes each governed by tightly regulated protein trafficking, signaling, and turnover. *Drosophila* PNs provided a powerful model system for us to systematically evaluate the neurodevelopmental functions of individual endosome-associated Rabs within the same cell types *in vivo.* Through this approach, we found that even within a single neuron, distinct post-endocytic sorting events regulate different aspects of development and, in some cases, act in a compartment specific manner (Figure 8). Below we discuss how studying membrane trafficking events *in vivo* can expand our understanding of neuronal development and cell biology.

Among the Rabs we tested, Rab5 regulated the broadest range of developmental events—likely reflecting its role in establishing the initial sorting hub from which many internalized proteins are routed. In contrast, Rab7 and Rab11 had more specialized roles with Rab11 directing a larger set of developmental processes than Rab7. Notably, Rab11 and Rab5 regulated largely non-overlapping processes, except for promoting axon branching and restraining bouton size in the mushroom body. These distinctions suggest that additional Rab-mediated trafficking routes downstream of early endosomes are also critical for circuit assembly. Thus, future studies defining how these additional pathways contribute to circuit assembly will be critical.

Despite extensive study, the role of Rab5 in neuritogenesis and growth remains debated, with some reports indicating that it inhibits these processes and others finding the opposite (Villarroel-Campos et al., 2016). These inconsistencies may stem from the reliance on overexpression systems, dominant-negative constructs, or *in vitro* models. Our clonal analysis approach enabled us to directly probe the role of Rab5 in circuit assembly. Our findings, which recapitulate those from a prior study (Sakuma et al., 2014), indicate that neurites still form and extend in the absence of Rab5, but both axons and dendrites fail to properly navigate to their appropriate targets. This is consistent with another report that found Rab5 is required for axon targeting of cortical neurons (Wu et al., 2014). Extending these insights, we defined additional roles for Rab5 and found that it is required to restrain PN axon branching and bouton size — processes that are distinct from initial neurite formation. We also showed that early endosomes are distributed in nascent boutons where they can exact their developmental functions. Collectively, our data highlight the pleiotropic roles of Rab5 in multiple neurodevelopmental processes.

Rab7 played a limited role in regulating PN dendrite targeting. Though Rab7 is the primary regulator of late endosome-lysosome fusion (Bucci et al., 2000; Vanlandingham & Ceresa, 2009; Xing et al., 2021), our data suggest that Rab7 plays a relatively minor role in PN development—consistent with prior studies of dendritic (Harish et al., 2019) and axonal (Ponomareva et al., 2016) branching. Rab7 did have non-autonomous effects on dendrite targeting for the PN types we examined. Perturbing Rab7 or endosomal acidification has been shown to cause a buildup of early endosomes (Girard et al., 2014; Lelouvier & Puertollano, 2011; Martina et al., 2009), raising the possibility that in some PNs impaired degradation of receptors could prolong endosomal signaling and lead to these non-autonomous effects on nearby neurons. This is in line with findings from embryonic development where Rab7 was shown to non-autonomously regulate receptor signaling during gastrulation (Kawamura et al., 2020).

A possible explanation for the absence of phenotypes upon Rab7 loss in single-cell clones is compensation by other Rabs. While Rab9 and Rab2 associate with degradative compartments, Rab9 is not expressed in developing PNs, making it unlikely to compensate for Rab7 loss (Figure 1-figure supplement 1A). Rab2 is expressed in PNs but primarily functions in autophagosome-lysosome fusion (Ding et al., 2019) and trafficking of lysosomes and their associated proteins toward late endosomes (Lund et al., 2018), rather than directing internalized cargo towards degradative compartments. Thus, while Rab2 is associated with the degradative pathway it does not have overlapping functions with Rab7. Our findings, together with prior studies inhibiting Rab7 function (Harish et al., 2019; Ponomareva et al., 2016), indicate that Rab7-mediated degradation is largely dispensable for circuit assembly, provided that upstream trafficking events adequately remove receptors from the plasma membrane and direct them to early endosomes.

Rab11, on the other hand, was important for axonal maturation particularly in the formation and stabilization of boutons and branches. Strikingly, even within the same axon, Rab11 regulated distinct aspects of development: in the mushroom body, Rab11 promoted axonal branching, whereas in the lateral horn, it was critical for the formation of certain branches and the stabilization of others. Given Rab11’s well-established role in endosomal recycling, these phenotypes are consistent with a failure to deliver adhesion and signaling receptors back to the plasma membrane—disrupting maturation of nascent axonal processes. Further, the compartment-specific effects of Rab11 (in mushroom body vs. lateral horn projections) suggests that distinct sets of cargos may be recycled in different axonal regions to drive local developmental processes.

Underscoring the specificity of endocytic recycling, Rab11 regulated distinct aspects of dendrite development across PN types. In DL1-PNs, Rab11-mediated recycling was critical for dendrites to fully innervate their target glomerulus, whereas this GTPase promoted dendrite targeting in DA1-PNs and VA1d/DC3-PNs. These findings not only extend the role for Rab11 beyond just dendrite branching (Takano et al., 2014) but also implies that it recycles a unique set of cargoes in each cell type. Supporting this idea, the Rab11-dependent DL1-PN dendrite phenotype, but not the VA1d-PN phenotype, resembles that observed upon loss of the cell adhesion protein Dscam, which normally promotes dendritic self-avoidance (Zhu et al., 2006). These data raise the possibility endocytic recycling regulates self-recognition events critical for dendrite elaboration, though further studies are needed to directly test this. Collectively, our findings highlight how endosomal recycling can exert cell type- and compartment-specific control over neuronal development. Defining the cargos regulated by Rab11-mediated trafficking will be essential for understanding how this pathway sculpts connectivity. Altogether, our work reveals how neurons deploy multiple endocytic routes in a compartment- and cell type–specific manner to direct morphogenesis and circuit development.

## MATERIALS AND METHODS

### Materials availability

We will deposit newly generated constructs in Addgene, and newly generated transgenic flies in the Bloomington Drosophila Stock Center. All other unique reagents generated in this study are available from the corresponding author (cnm@stanford.edu or lluo@stanford.edu)

### *Drosophila* stocks and husbandry

Flies were maintained on standard cornmeal media with a 12 hr light-dark cycle at 25°C, except for overexpression crosses which were raised at 29°C. The strains used are described in the Key Resources Table and complete genotypes for flies used in each figure are in Table S1.

### Generation of UAS/QUAS constructs and transgenic flies

The *UAS-mCherry-Rab7* and *QUAS-mCherry-Rab7* constructs were synthesized and cloned by Twist Biosciences into a 10x pUASt-attB vector or 10x QUAS-attB vector. The mCherry tags are N-terminal to preserve Rab function. The construct was validated by full-length plasmid sequencing and injected into embryos with an attP40 insertion site. G0 flies were crossed to a white− balancer and all white+ progeny were individually balanced. Flies were injected in-house using standard microinjection methods.

### Immunofluorescence staining and confocal microscopy

Fly brains were dissected according to a previously published protocol (J. S. Wu & Luo, 2006a). In brief, brains were dissected in PBS, transferred to a tube containing 4% paraformaldehyde in PBST (0.3% Triton X-100), and fixed for 20 min while nutating at RT. Following fixation, brains were washed 3 times for 20 minutes in PBST and blocked for at least 30 min in PBST + 5% normal donkey serum. The following antibodies were used: rat anti-Ncad (Developmental Studies Hybridoma Bank [DSHB]; 1:40), chicken anti-GFP (Aves Labs; 1:1000), rabbit anti-dsRed (Takara Bio; 1:1000), mouse anti-bruchpilot (DSHB; 1:100), mouse anti-mCherry (ThermoFisher Scientific; 1:1000) and incubated with brains in block buffer overnight at 4°C while nutating. Brains were subsequently washed three times for 20 min in PBST and incubated in secondary antibodies (Alexa Fluor 488; Alexa Fluor 564; Alexa Fluor 647; 1:200) overnight at 4°C while nutating. Brains were again washed three times for 20 min in PBST, transferred to SlowFade antifade reagent (ThermoFisher) and stored at 4°C prior to mounting.

### Image acquisition and processing

Images were obtained on a Zeiss LSM900 laser-scanning confocal microscope (Carl Zeiss) using either a 40x oil immersion objective (dendrite targeting and axon morphology experiments) or a 63x oil immersion objective (Airyscan experiments). 16-bit z-stacks for dendrite targeting and axon morphology experiments were acquired at 1 μm intervals at a resolution of 1024 × 1024. Airyscan images were taken at software optimized resolution and intervals. Brightness and contrast adjustments as well as image cropping was done using Photoshop or Illustrator (Adobe).

### Dominant negative screen

Virgin *Drosophila* females with the genotype *VT033006-GAL4, GMR86C10-LexA>LexAop-mtdTomato, Or98a-mC-D8::GFP, Or92a-CD2* were crossed with *UAS-RabX-DN* males to express GDP-locked dominant negative Rabs in *VT-GAL4* PNs, and the progeny were kept at 25°C for 2–5 days following egg laying and then transferred to 29°C to enhance transgene expression. Brains were dissected, processed, and imaged as described above. See Table S1 for complete genotypes.

For this analysis, we identified glomeruli using NCad labeling (based on the stereotypy of their size, shape, and positions). VM5d/v PNs were monitored through the expression of mtdTomato using the *GMR86C10-LexA* driver, and dendrite targeting was categorized by the presence or absence of mistargeting to ectopic glomeruli. Dendrite targeting analysis was performed blinded to genotype when possible. Fisher’s exact test was performed on mistargeting frequencies to determine statistical significance compared to controls. P-values were adjusted using the Benjamini-Hochberg procedure.

### MARCM-based clonal analyses

Clonal analyses using mosaic analysis with a repressible cell marker (MARCM) and Q-MARCM (MARCM using the Q system) have been previously described (Potter & Luo, 2011; J. S. Wu & Luo, 2006b). Each fly contains a *hsFLP122* recombinase, *GH146-GAL4* (PN GAL4) or *GH146-QF* (PN QF), *TubP*-*Gal80* or *TubP*-*QS*, *UAS-mCD8-GFP* or *QUAS-mCD8-GFP*, the desired *FRT*, and either wild-type or a mutant allele distal to the *FRT* site; flies for the Rab11 rescue experiments also included a *UAS-mCherry-Rab11*, and flies for the Rab7 rescue experiments included a *QUAS-mCherry-Rab7* (see Table S1 for complete genotypes). To generate adPN neuroblast, DL1 single-cell, DA1 neuroblast, and VA1d/DC3 neuroblast clones, flies were heat shocked for 1 hour at 37 °C at 0–24h after larval hatching. To generate smaller neuroblast clones with projections to the DM6 glomerulus, flies were heat shocked for 1 hour at 37 °C at 48–72h after larval hatching. Brains were dissected and processed as described above. We identified glomeruli using NCad labeling and categorized the extent of innervation into each glomerulus (not innervated; weakly innervated; moderately innervated; strongly innervated). Analysis was performed blinded to experimental manipulation. For each glomerulus, we calculated the frequency of each type of innervation and plotted the results as stacked bar charts. Fisher’s exact test was performed on innervation frequencies in each glomerulus, using counts for wildtype levels of innervation (strong if innervation is expected and none if innervation is not expected in wildtype) vs. non-wildtype levels of innervation, to determine statistical significance compared to controls. P-values were adjusted using the Benjamini-Hochberg test.

### Image analysis and quantification

Microscopy images were processed and analyzed using ImageJ tools. Dendrite glomerular innervation was measured using the area measuring tool where the area of each DL1 glomerulus was measured using NCad staining and compared to the area of GFP-positive dendrites within that glomerulus. Bouton diameter was measured by drawing a line across the widest part of each bouton and measuring the length using the length measuring tool. Axon branches were traced and measured using Semi-automated Tracing on GFP-positive axons in the Simple Neurite Tracer (SNT) plugin (Arshadi et al., 2021) to quantify branch numbers and lengths. All analyses were done blinded to genotype.

Volume renderings were created using Imaris10 (Oxford Instruments); Airyscan super-resolution images (Carl Zeiss) were imported, and the Surfaces tool was used to model the membrane of GFP-positive dendrites and axons as well as mask the puncta channel. The Surfaces tool was used to model puncta with the masked puncta channel, and the Spots tool was used to quantify the number of puncta. Thresholds were set manually.

### Statistical analysis

Statistical comparisons were done as such: Fisher’s exact test was used for categorical data (e.g. mistargeting, neuroblast glomerular innervation); Mann-Whitney U test was used to compare quantitative data between two groups (e.g. bouton diameter between controls and mutants); Kruskal-Wallis test was used to compare quantitative data between three groups (e.g. bouton diameter between controls, mutants, and rescues). The numbers of independent replicates per experiment are indicated in the figures or legends.

## Supplementary Material

Supplement 1

## Figures and Tables

**Key Resources Table:** 

Reagent type (species) or resource	Designation	Source or reference	Identifiers	Additional information
Genetic reagent (*D. melanogaster*)	*VT033006-GAL4*	DOI: 10.1101/198648 (Tirian & Dickson, 2017)	RRID:BDSC_73333	
Genetic reagent (*D. melanogaster*)	*GMR86C10-LexA*	DOI: 10.1073/pnas.1905832116 (Xie et al., 2019)	N/A	
Genetic reagent (*D. melanogaster*)	*LexAop-mtdTomato*	DOI: 10.1073/pnas.1905832116 (Xie et al., 2019)	N/A	
Genetic reagent (*D. melanogaster*)	*Or98a-mCD8-GFP*	DOI: 10.1016/j.cub.2005.07.034 (Couto et al., 2005)	RRID:BDSC_52647	
Genetic reagent (*D. melanogaster*)	*Or92a-rCD2*	DOI: 10.7554/eLife.39088 (Li et al., 2018)	N/A	
Genetic reagent (*D. melanogaster*)	*UAS-lacZ*	DOI: 10.1242/dev.118.2.401 (Brand & Perrimon, 1993)	RRID:BDSC_1776	
Genetic reagent (*D. melanogaster*)	*UAS-Rab5.S43N*	Bloomington *Drosophila* Stock Center	RRID:BDSC_42703	Bellen, 2012
Genetic reagent (*D. melanogaster*)	*UAS-Rab5.S43N*	Bloomington *Drosophila* Stock Center	RRID:BDSC_42704	Bellen, 2012
Genetic reagent (*D. melanogaster*)	*UASp-YFP.Rab11.S25N*	DOI: 10.1534/genetics.106.066761 (Zhang et al., 2007)	RRID:BDSC_23261	
Genetic reagent (*D. melanogaster*)	*UASp-YFP.Rab11.S25N*	DOI: 10.1534/genetics.106.066761 (Zhang et al., 2007)	RRID:BDSC_9792	
Genetic reagent (*D. melanogaster*)	*UASp-YFP.Rab7.T22N*	DOI: 10.1534/genetics.106.066761 (Zhang et al., 2007)	RRID:BDSC_9778	
Genetic reagent (*D. melanogaster*)	*UAS-mCD8-GFP*	DOI: 10.1016/S0896-6273(00)80701-1 (Lee & Luo, 1999)	RRID:DG-GR_108068	
Genetic reagent (*D. melanogaster*)	*hsFlp122*	DOI: 10.1016/0092-8674(93)90072-X (Struhl & Basler, 1993)	N/A	
Genetic reagent (*D. melanogaster*)	*TubP-Gal80*	DOI: 10.1016/S0896-6273(00)80701-1 (Lee & Luo, 1999)	RRID:BDSC_9917	
Genetic reagent (*D. melanogaster*)	*GH146-GAL4*	DOI: 10.1002/(SICI)1097-4695(199705)32:5<443::AID-NEU1>3.0.CO;2-5 (Stocker et al., 1997)	RRID:BDSC_30026	
Genetic reagent (*D. melanogaster*)	*Rab5^2^*	DOI: 10.1083/jcb.200211087 (Wucherpfennig et al., 2003)	"N/A	
Genetic reagent (*D. melanogaster*)	*Rab11^EP3017^*	DOI: 10.1534/genetics.104.026427 (Bellen et al., 2004)		
Genetic reagent (*D. melanogaster*)	*UAS-mCherry-Rab11*	DOI: 10.1083/jcb.202103069 (W. Chen & He, 2022)	RRID:BDSC_42708	
Genetic reagent (*D. melanogaster*)	*FRT40A*	DOI: 10.1242/dev.117.4.1223 (Xu & Rubin, 1993)	N/A	
Genetic reagent (*D. melanogaster*)	*FRT82B*	DOI: 10.1242/dev.117.4.1223 (Xu & Rubin, 1993)	N/A	
Genetic reagent (*D. melanogaster*)	*FRT19A*	DOI: 10.1242/dev.117.4.1223 (Xu & Rubin, 1993)	N/A	
Genetic reagent (*D. melanogaster*)	*GH146-QF*	DOI: 10.1016/j.cell.2010.02.025 (Potter et al., 2010)	RRID:BDSC_1744	
Genetic reagent (*D. melanogaster*)	*TubP-QS*	DOI: 10.1016/j.cell.2010.02.025 (Potter et al., 2010)	RRID:BDSC_30015	
Genetic reagent (*D. melanogaster*)	*QUAS-mCD8-GFP*	DOI: 10.1016/j.cell.2010.02.025 (Potter et al., 2010)	RRID:BDSC_30022	
Genetic reagent (*D. melanogaster*)	*QUAS-mCherry-Rab7*	This paper	RRID:BDSC_30001	
Genetic reagent (*D. melanogaster*)	*UAS-mCherry-Rab7*	This paper	N/A	
Genetic reagent (*D. melanogaster*)	*UAS-2xFYVE-mCherry*	DOI: 10.1101/2025.08.28.672852 (McLaughlin et al., 2025)	N/A	
Genetic reagent (*D. melanogaster*)	*Rab7^GAL4-KO^*	DOI: 10.7554/eLife.01064 (Cherry et al., 2013)	N/A	
Genetic reagent (*D. melanogaster*)	*MZ19-GAL4*	DOI: 10.1101/lm.5.1.52 (Ito et al., 1998)	N/A	
Genetic reagent (*D. melanogaster*)	*UAS-FRT10-stop-FRT10-3xHalo7-CAAX*	DOI: 10.7554/eLife.85521 (Wong et al., 2023)	RRID:BDSC_34497	
Genetic reagent (*D. melanogaster*)	*71B05-GAL4*	DOI: 10.1016/j.celrep.2012.09.011 (Jenett et al., 2012)	N/A	
Antibody	Chicken anti-GFP	Aves Labs Inc	N/A	
Antibody	Rat anti-NCad	Developmental Studies Hybridoma Bank	RRID:AB_10000240	(1 :1000 in 5% normal donkey serum)
Antibody	Rabbit anti-DsRed	ClontechTakara	RRID:AB_528121	(1 :40 in 5% normal donkey serum)
Antibody	Mouse anti-bruchpilot	Developmental Studies Hybridoma Bank	RRID:AB_10013483	(1 :1000 in 5% normal donkey serum)
Antibody	Rat anti-mCherry	Thermo Fischer	RRID:AB_2314866	(1 :100 in 5% normal donkey semm)
Antibody	Alexa Fluor 488 AffiniPure Donkey Anti-Chicken	Jackson Immunoresearch	RRID:AB_2536611	(1 :500 in 5% normal donkey serum)
Antibody	Cy3 AffiniPure Donkey Anti-Rabbit	Jackson Immunoresearch	RRID:AB_2340375	(1 :200 in 5% normal donkey semm)
Antibody	Alexa Fluor 647 AffiniPure Donkey Anti-Rat	Jackson Immunoresearch	RRID:AB_2307443	(1 :200 in 5% normal donkey serum)
Antibody	AMCA AffiniPure Donkey Anti-Mouse	Jackson Immunoresearch	RRID:AB_2340694	(1 :200 in 5% normal donkey semm)
Antibody	DyLight 405 AffiniPure Donkey Anti-Rat	Jackson Immunoresearch	RRID:AB_2340806	(1 :200 in 5% normal donkey serum)
Software, algorithm	ZEN	Carl Zeiss	RRID:AB_2340681	(1 :200 in 5% normal donkey serum)
Software, algorithm	Fiji	National Institutes of Health	RRID:SCR_013672	
Software, algorithm	SNT	Arshadi et al., 2021(Arshadi et al., 2021)	RRID:SCR_002285	
Software, algorithm	Illustrator	Adobe	N/A	
Software, algorithm	Photoshop	Adobe	RRID:SCR_010279	
Software, algorithm	Imaris	Oxford Instruments	RRID:SCR_014199	
Software, algorithm	R Project for Statistical Computing	N/A	RRID:SCR_007370	http://www.bit-plane.com/imaris/imaris
			RRID:SCR_001905	http://www.r-project.org/
