# Supplementary material for: Endosome-associated Rab GTPases control distinct aspects of neural circuit assembly
Source: bioRxiv. 2025 Oct 10:2025.10.09.681358. Preprint. [Version 1] doi: 10.1101/2025.10.09.681358 (PMC12632621; doi:10.1101/2025.10.09.681358)
Supplement: Supplement 1 [file NIHPP2025.10.09.681358v1-supplement-1.pdf]

bioRxiv preprint doi: https://doi.org/10.1101/2025.10.09.681358; this version posted October 10, 2025. The copyright holder for this preprint (which was not certified by peer review) is the author/funder, who has granted bioRxiv a license to display the preprint in perpetuity. It is made available under aCC-BY 4.0 International license.

Supplemental Material for:

# Endosome-associated Rab GTPases control distinct aspects of neural circuit assembly

Katherine X. Dong, Hui Ji, David J. Luginbuhl, Liqun Luo, Colleen N. McLaughlin

bioRxiv preprint doi: https://doi.org/10.1101/2025.10.09.681358; this version posted October 10, 2025. The copyright holder for this preprint (which was not certified by peer review) is the author/funder, who has granted bioRxiv a license to display the preprint in perpetuity. It is made available under aCC-BY 4.0 International license.

bioRxiv preprint doi: https://doi.org/10.1101/2025.10.09.681358; this version posted October 10, 2025. The copyright holder for this preprint (which was not certified by peer review) is the author/funder, who has granted bioRxiv a license to display the preprint in perpetuity. It is made available under aCC-BY 4.0 International license.

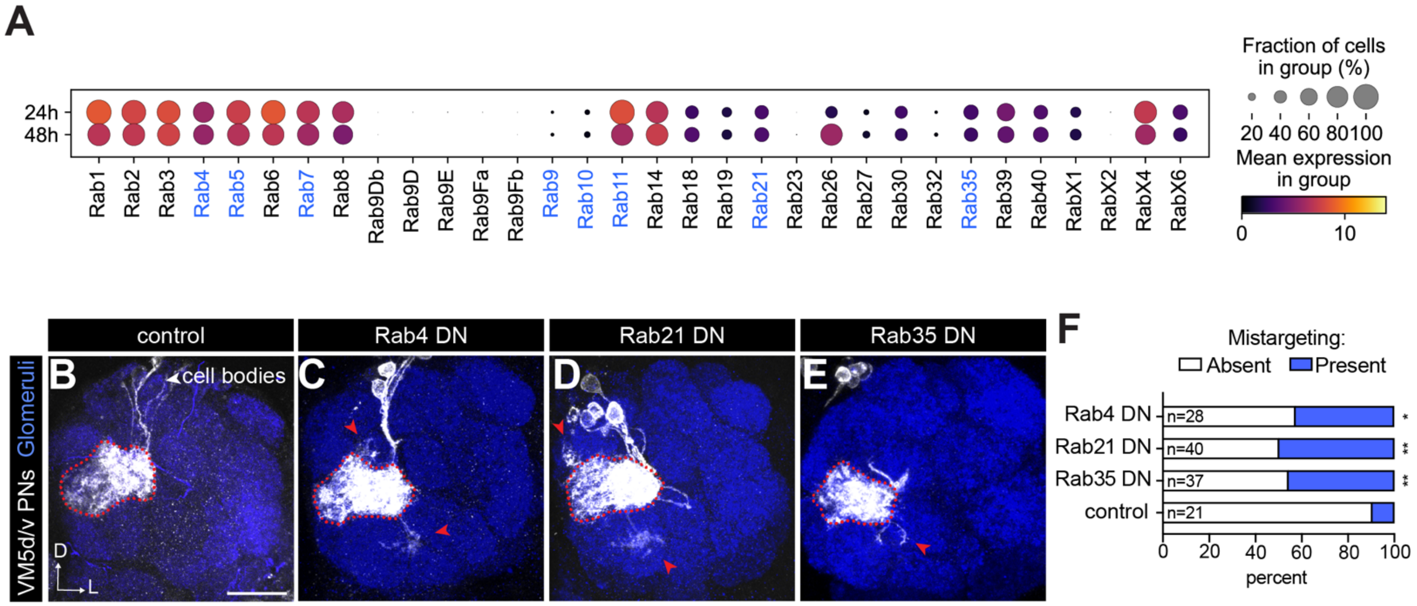


**Figure 1-figure supplement 1. Extend analysis of endosome-associated Rabs.**
(A) Expression of Rab GTPases in developing PNs at 24h after puparium formation (APF) and 48h APF from single-cell RNA-seq (scRNA-seq) data. Endosome-associated Rabs are colored blue. Expression is in $\log_2$(CPM +1), where CPM stands for transcript counts per million reads. scRNA-seq data are from Xie et al., 2021.
(B–E) Representative images of indicated genotypes depicting phenotypes observed in dominant negative screen. Red dotted lines outline the VM5d/v glomeruli, and red arrows denote ectopic targeting. Scale bar, 20 µm.
(F) Percent of antennal lobes with mistargeting in the Rab dominant negative screen.

bioRxiv preprint doi: https://doi.org/10.1101/2025.10.09.681358; this version posted October 10, 2025. The copyright holder for this preprint (which was not certified by peer review) is the author/funder, who has granted bioRxiv a license to display the preprint in perpetuity. It is made available under aCC-BY 4.0 International license.

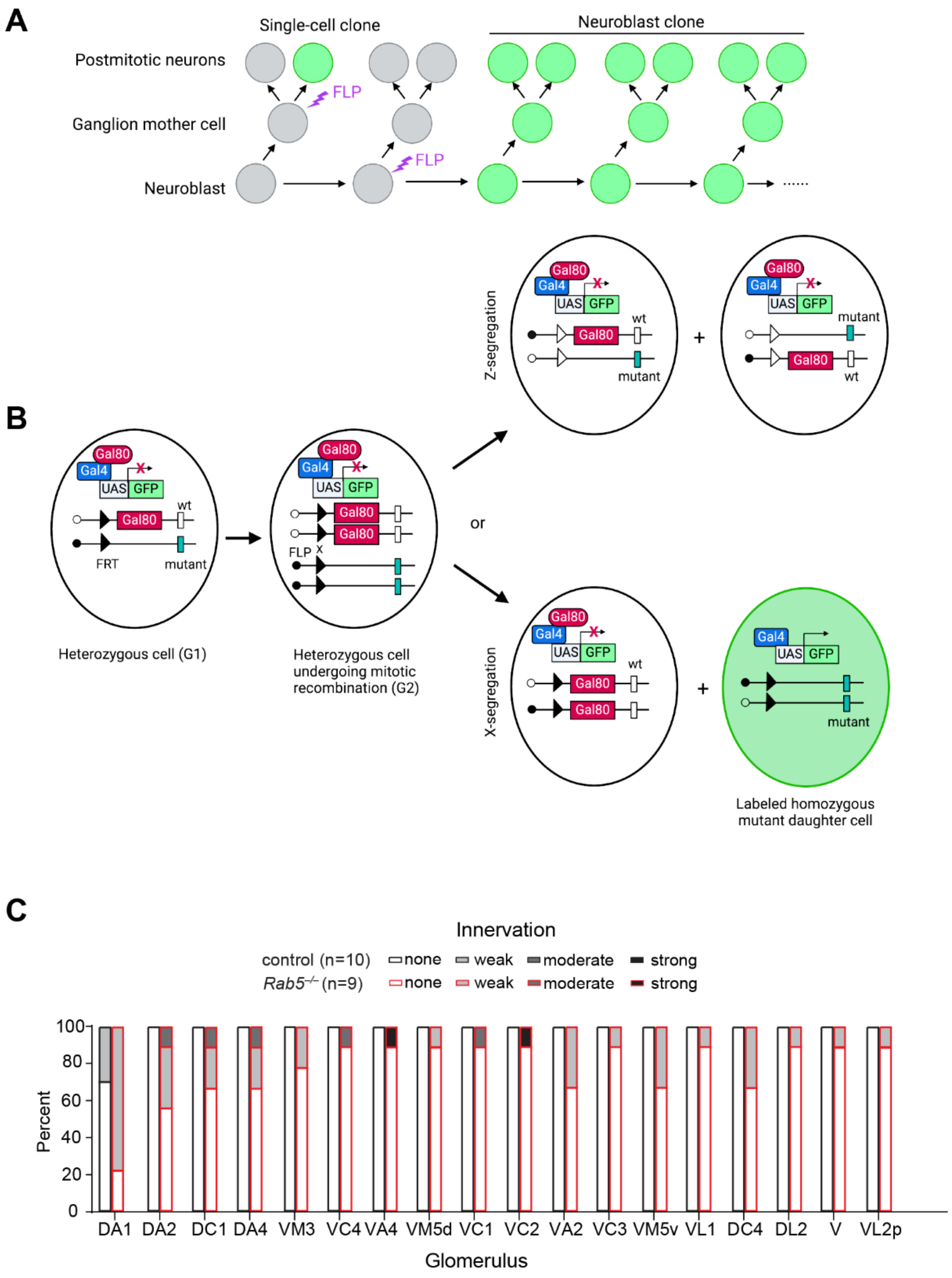


**Figure 2-figure supplement 1. Schematic of MARCM-based mosaic analysis.**
(A) Schematic depicting heatshock flp induction of single-cell or neuroblast MARCM clones. MARCM can be used to generate GFP-labeled single-cell or neuroblast clones in PNs. All clones were induced by heat shock applied to newly hatched larvae (0–24h after larval hatching), so our analyses are primarily restricted to the adPNs and DL1-PN single-cell clones.
(B) Schematic of MARCM analysis. A mutant (*Rab5*, *Rab7*, or *Rab11*) allele is placed on a chromosome arm in *trans* to the chromosome arm with a *GAL80* transgene. Heterozygous cells express GAL80, which represses GAL4 activity and thus inhibits GFP expression in these cells. Following, FLP-mediated mitotic recombination and X-segregation (bottom row) one of the daughter cells becomes homozygous for the mutant allele and loses the *GAL80* transgene. Thus, homozygous mutant cells will be labeled with membrane-bound GFP (and can also express any UAS-based rescue transgene).
(C) Quantification of adPN mistargeting in additional non-adPN glomeruli.

bioRxiv preprint doi: https://doi.org/10.1101/2025.10.09.681358; this version posted October 10, 2025. The copyright holder for this preprint (which was not certified by peer review) is the author/funder, who has granted bioRxiv a license to display the preprint in perpetuity. It is made available under aCC-BY 4.0 International license.

bioRxiv preprint doi: https://doi.org/10.1101/2025.10.09.681358; this version posted October 10, 2025. The copyright holder for this preprint (which was not certified by peer review) is the author/funder, who has granted bioRxiv a license to display the preprint in perpetuity. It is made available under aCC-BY 4.0 International license.

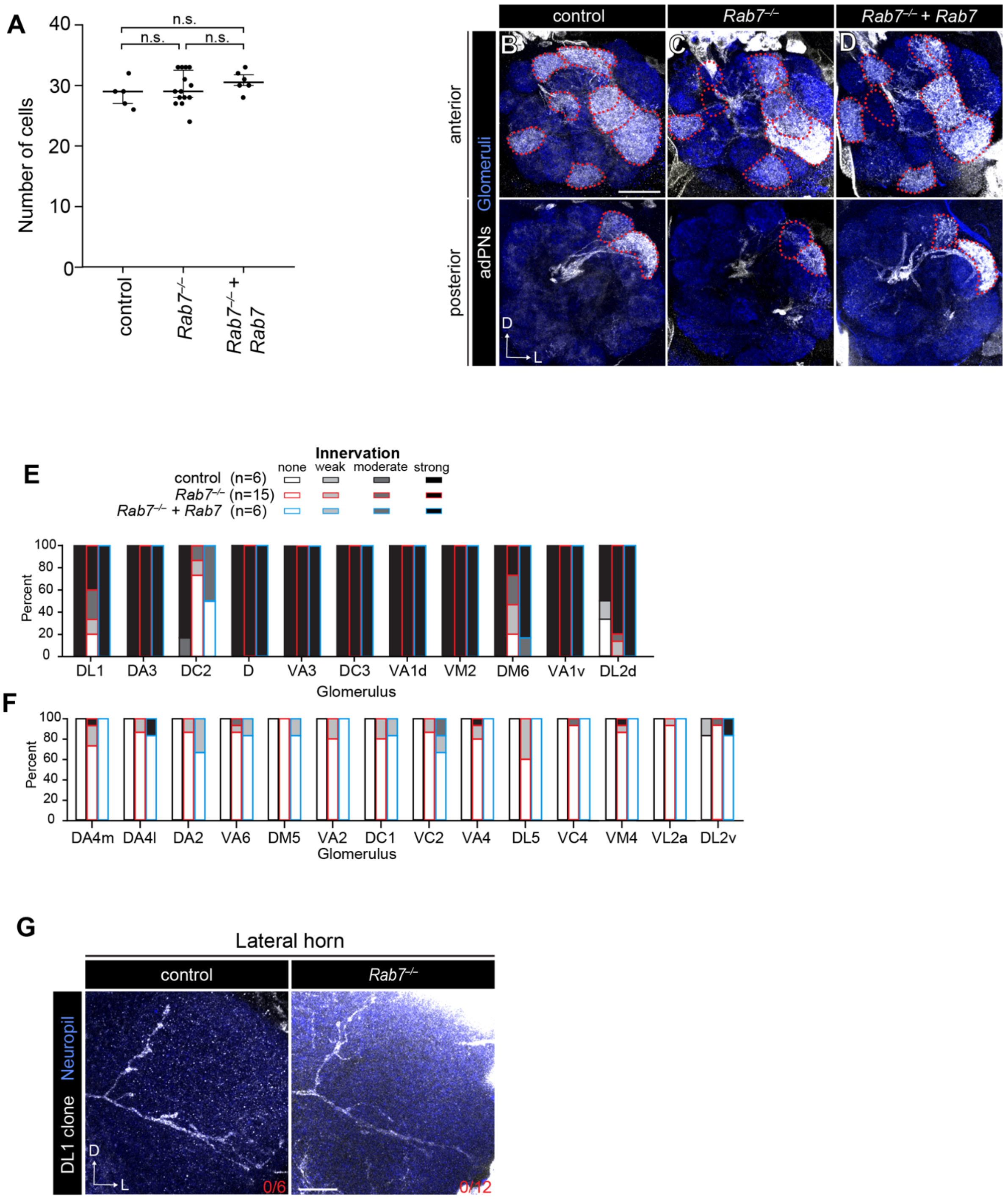


**Figure 4-figure supplement 1. Extended analysis of *Rab7* loss-of-function MARCM phenotypes.**
(A) Quantification of number of cell bodies in adPN neuroblast clones of controls (n=5), *Rab7*$^{-/-}$ mutants (n=14), and *Rab7* rescues (n=6).
(B–D) Representative images of adPN neuroblast clones of indicated genotypes.
(E, F) Quantification of percent of antennal lobes with each category of dendrite innervation to adPN glomeruli (E) and non-adPN glomeruli (F) in indicated genotypes.
(G) Representative images of DL1-PN lateral horn axons in indicated genotypes.

Scale bar, 20 μm (B), 10 μm (G).

bioRxiv preprint doi: https://doi.org/10.1101/2025.10.09.681358; this version posted October 10, 2025. The copyright holder for this preprint (which was not certified by peer review) is the author/funder, who has granted bioRxiv a license to display the preprint in perpetuity. It is made available under aCC-BY 4.0 International license.

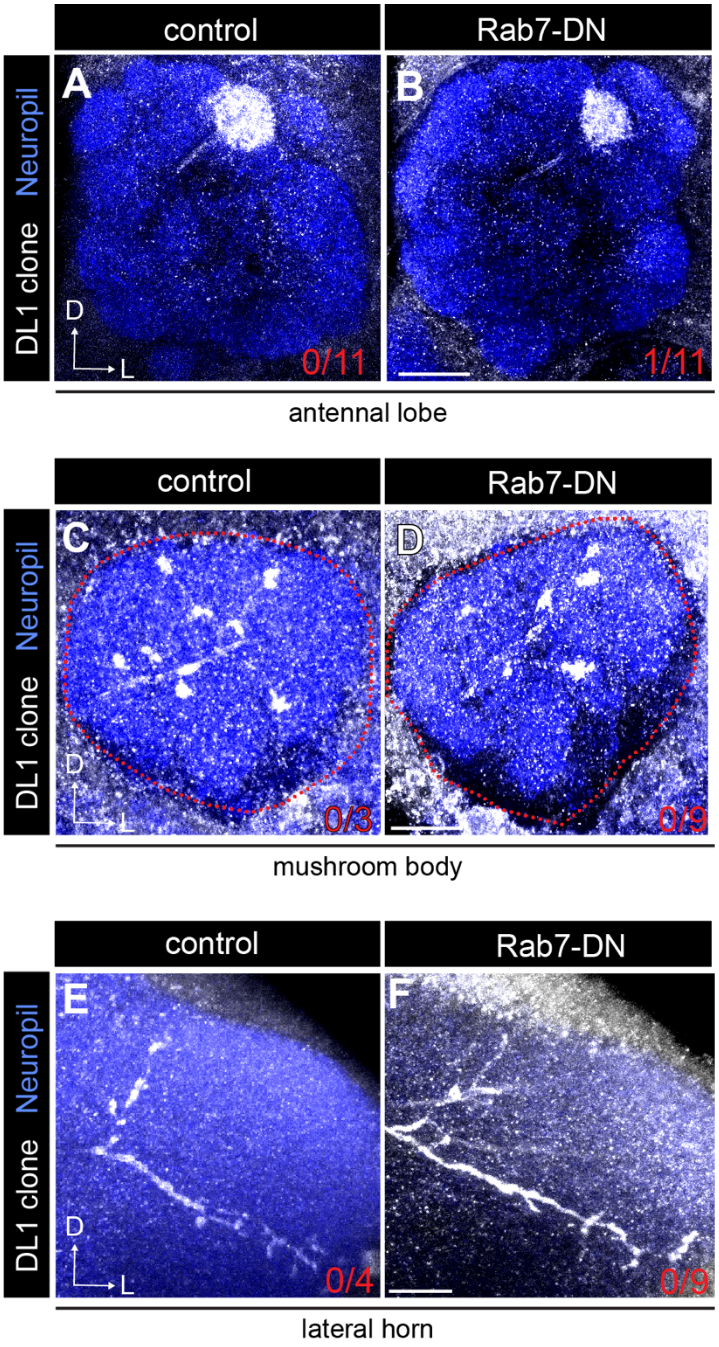


**Figure 4-figure supplement 2. Rab7 is dispensable for DL1-PN development.**
(A, B) Representative images of DL1-PN dendrite targeting in indicated genotypes. Red numbers in the right corner of images denote DL1-PN dendrite mistargeting phenotypic penetrance.
(C, D) Representative images of mushroom body axons in indicated genotypes. Red numbers in the right corner of images denote DL1-PN axon morphogenesis phenotypic penetrance.
(E, F) Representative images of lateral axons in indicated genotypes. Red numbers in the right corner of images denote DL1-PN axon morphogenesis phenotypic penetrance.

Scale bar, 20 µm (C); 10 µm (D, I)

bioRxiv preprint doi: https://doi.org/10.1101/2025.10.09.681358; this version posted October 10, 2025. The copyright holder for this preprint (which was not certified by peer review) is the author/funder, who has granted bioRxiv a license to display the preprint in perpetuity. It is made available under aCC-BY 4.0 International license.

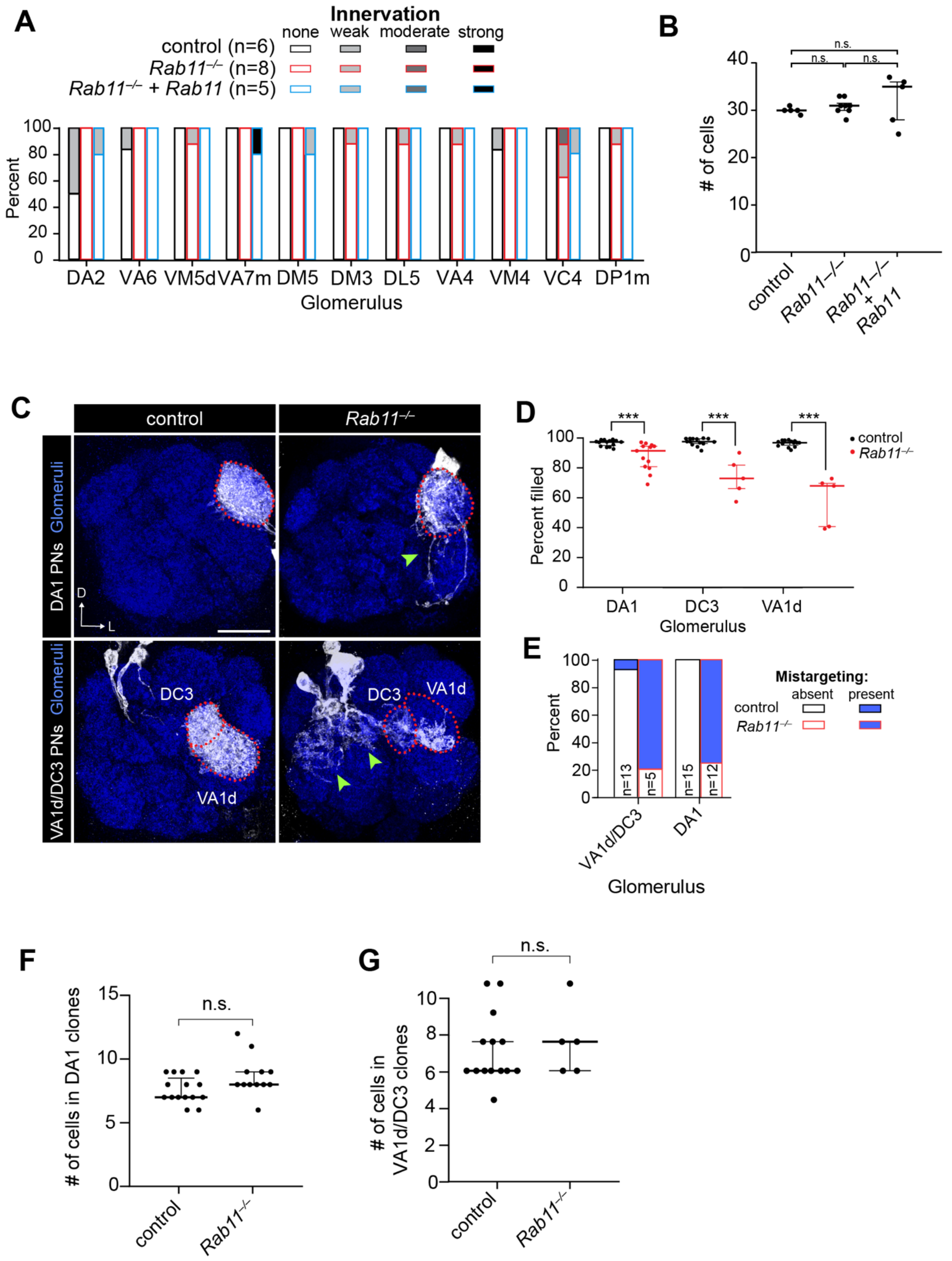


**Figure 5-figure supplement 1. Additional analysis of *Rab11* dendrite targeting phenotypes.**
(A) Quantification of ectopic dendrite targeting phenotypes across all analyzed non-adPN glomeruli, excluding those present in Figure 5E. Note the non-adPN glomeruli present in Figure 5E are significantly different.
(B) Quantification of number of cell bodies in adPN neuroblast clones of controls (n=5), *Rab11*$^{-/-}$ mutants (n=8), and *Rab11* rescues (n=5).

bioRxiv preprint doi: https://doi.org/10.1101/2025.10.09.681358; this version posted October 10, 2025. The copyright holder for this preprint (which was not certified by peer review) is the author/funder, who has granted bioRxiv a license to display the preprint in perpetuity. It is made available under aCC-BY 4.0 International license.

(C) Representative images of mistargeting observed in indicated genotypes. Red dotted outline denotes DA1 (top row) or DC3/VA1d (bottom row) glomeruli. Green arrows denote mistargeting.
(D) Quantification of the percent of the indicated glomerulus filled with PN dendrites in DA1-PN controls (n=15) and *Rab11*$^{-/-}$ mutants (n=12) and VA1d/DC3-PN controls (n=14) and *Rab11*$^{-/-}$ mutants (n=5).
(E) Quantification of the proportion of antennal lobes with mistargeting.
(F) Quantification of the number of cell bodies in neuroblast clones containing labeled DA1-PNs visualized by the MZ19-GAL4 driver for controls (n=15) and *Rab11*$^{-/-}$ mutants (n=12).
(G) Quantification of the number of cell bodies in neuroblast clones containing labeled VA1d/DC3-PNs visualized by the MZ19-GAL4 driver controls (n=14) and *Rab11*$^{-/-}$ mutants (n=5).

Scale bar, 20 µm (C).

bioRxiv preprint doi: https://doi.org/10.1101/2025.10.09.681358; this version posted October 10, 2025. The copyright holder for this preprint (which was not certified by peer review) is the author/funder, who has granted bioRxiv a license to display the preprint in perpetuity. It is made available under aCC-BY 4.0 International license.

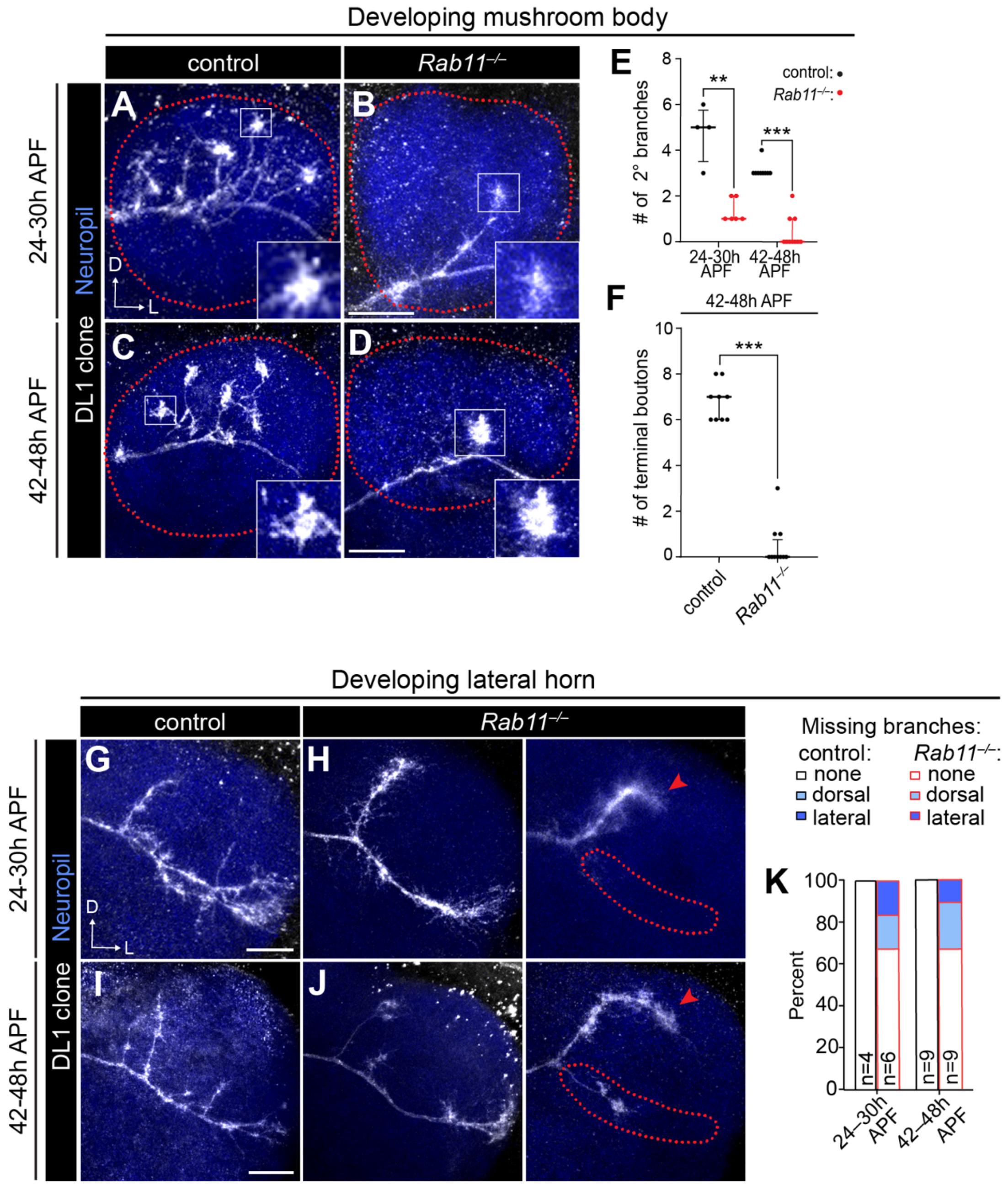


**Figure 6-figure supplement 1. Extended analysis of *Rab11* axon development phenotypes.**
(A–D) Representative images of control and *Rab11*$^{-/-}$ mutant DL1-PN axons in the mushroom body at 24-30h and 42-48h APF. Dotted lines denote the border of the mushroom body.
(E) Quantification of the number of secondary branches in each DL1-PN axon at the mushroom body for 24-30h APF controls (n=4) and *Rab11*$^{-/-}$ mutants (n=6) and 42-48h APF controls (n=9) and Rab*11*$^{-/-}$ mutants (n=10).
(F) Quantification of the number of terminal boutons in each DL1-PN axon at the mushroom body for 42-48h APF controls (n=9) and Rab*11*$^{-/-}$ mutants (n=10).
(G–J) Representative images of control and Rab*11*$^{-/-}$ mutant DL1-PN axons in the lateral horn at 24–30h and 42–48h APF. Dotted lines denote the border of the loss of lateral branches. Arrow heads indicate overextension of the dorsal branch.
(K) Quantification of the percentage of lateral horn axons that have missing branches in each genotype at indicated developmental timepoints.

Scale bars, 10 µm.

bioRxiv preprint doi: https://doi.org/10.1101/2025.10.09.681358; this version posted October 10, 2025. The copyright holder for this preprint (which was not certified by peer review) is the author/funder, who has granted bioRxiv a license to display the preprint in perpetuity. It is made available under aCC-BY 4.0 International license.

**Table S1. *Drosophila* genotypes in each figure.**

| Figure | Genotype |
|---|---|
| 1E-I | **Control**: *UAS-lacZ /+; VT033006-GAL4, GMR86C10-LexA, LexAop-mtdTomato, Or98a-mCD8-GFP, Or92a-CD2/+*<br>**Rab5 DN #1**: *UAS-Rab5.S43N* (BDSC 42703) /+; *VT033006-GAL4, GMR86C10-LexA>LexAop-mtdTomato, Or98a-mCD8-GFP, Or92a-CD2/+*<br>**Rab5 DN #2**: *VT033006-GAL4, GMR86C10-LexA, LexAop-mtdTomato, Or98a-mCD8-GFP, Or92a-CD2/UAS-Rab5.S43N* (BDSC 42704)<br>**Rab11 DN #1**: *VT033006-GAL4, GMR86C10-LexA, LexAop-mtdTomato, Or98a-mCD8-GFP, Or92a-CD2/UASp-YFP.Rab11.S25N* (BDSC 23261)<br>**Rab11 DN #2**: *UASp-YFP.Rab11.S25N* (BDSC 9792) /+; *VT033006-GAL4, GMR86C10-LexA, LexAop-mtdTomato, Or98a-mCD8-GFP, Or92a-CD2/+*<br>**Rab7 DN**: *VT033006-GAL4, GMR86C10-LexA>LexAop-mtdTomato, Or98a-mCD8-GFP, Or92a-CD2/UASp-YFP.Rab7.T22N* (BDSC 9778) |
| 1-figure supplement 1B-F | **Control**: *UAS-lacZ /+; VT033006-GAL4, GMR86C10-LexA, LexAop-mtdTomato, Or98a-mCD8-GFP, Or92a-CD2/+*<br>**Rab4 DN:** *UASp-YFP.Rab4.S22N* (BDSC 9768)/+; *VT033006-GAL4, GMR86C10-LexA, LexAop-mtdTomato, Or98a-mCD8-GFP, Or92a-CD2/+*<br>**Rab21 DN:** *UAST-YFP.Rab21.T27N* (BDSC 23241)/+; *VT033006-GAL4, GMR86C10-LexA, LexAop-mtdTomato, Or98a-mCD8-GFP, Or92a-CD2/+*<br>**Rab35 DN:** *UAST-YFP.Rab35.S22N* (BDSC 9819)/+; *VT033006-GAL4, GMR86C10-LexA, LexAop-mtdTomato, Or98a-mCD8-GFP, Or92a-CD2/+* |
| 2B-H<br>2-figure supplement 1C<br>3B-L | **Control**: *UAS-mCD8-GFP, hsFlp122/+* (or *Y*); *TubP-Gal80, FRT40A, GH146-GAL4, UAS-mCD8-GFP/FRT40A*<br>***Rab5*** $^{-/-}$: *UAS-mCD8-GFP, hsFlp122/+* (or *Y*); *TubP-Gal80, FRT40A, GH146-GAL4, UAS-mCD8-GFP/ Rab5* $^{2}$*, FRT40A* |
| 2I, J<br>3M-P | *UAS-mCD8-GFP, hsFlp122/+* (or *Y*); *TubP-Gal80, FRT40A, GH146-GAL4, UAS-mCD8-GFP/FRT40A; UAS-2xFYVE-mCherry/+* |
| 4<br>4-figure supplement 1 | **Control**: *hsFlp122, QUAS-mCD8-GFP/+* (or *Y*);; *GH146-QF, FRT82B, TubP-QS/FRT82B*<br>***Rab7*** $^{-/-}$: *hsFlp122, QUAS-mCD8-GFP/+* (or *Y*);; *GH146-QF, FRT82B, TubP-QS/FRT82B, Rab7* $^{GAL4\text{-}KO}$<br>***Rab7*** $^{-/-}$**+*Rab7***: *hsFlp122, QUAS-mCD8-GFP/+* (or *Y*); *QUAS-mCherry-Rab7/+; GH146-QF, FRT82B, TubP-QS/FRT82B, Rab7* $^{GAL4\text{-}KO}$<br>**Rab7 OE:** *UAS-mCD8-GFP, hsFlp122/+* (or *Y*); *TubP-Gal80, FRT40A, GH146-GAL4, UAS-mCD8-GFP/FRT40A; UAS-mCherry-Rab7/+* |

bioRxiv preprint doi: https://doi.org/10.1101/2025.10.09.681358; this version posted October 10, 2025. The copyright holder for this preprint (which was not certified by peer review) is the author/funder, who has granted bioRxiv a license to display the preprint in perpetuity. It is made available under aCC-BY 4.0 International license.

| | |
|---|---|
| 4-figure supplement 2 | **Control**: *GH146-FLP/UAS-FRT10-stop-FRT10-3xHalo7-CAAX; 71B05-GAL4/UAS-lacZ*<br>**Rab7-DN**: *GH146-FLP/UAS-FRT10-stop-FRT10-3xHalo7-CAAX; 71B05-GAL4/UASp-YFP.Rab7.T22N* (BDSC 9778) |
| 5<br>5-figure supplement 1A, B<br>6<br>6-figure supplement 1<br>7 | **Control**: *UAS-mCD8-GFP, hsFlp122*; *GH146-GAL4/+*; *FRT82B, TubP-Gal80/FRT82B*<br>***Rab11*$^{-/-}$**: *UAS-mCD8-GFP, hsFlp122*; *GH146-GAL4/+*; *FRT82B, TubP-Gal80/FRT82B, Rab11*$^{EP3017}$<br>***Rab11*$^{-/-}$+*Rab11***: *UAS-mCD8-GFP, hsFlp122*; *GH146-GAL4/UAS-mCherry-Rab11*; *FRT82B, TubP-Gal80/FRT82B, Rab11*$^{EP3017}$ |
| 5-figure supplement 1C-G | **Control**: *UAS-mCD8-GFP, hsFlp122*; *MZ19-GAL4, UAS-mCD8-GFP/+*; *FRT82B, TubP-Gal80/FRT82B*<br>***Rab11*$^{-/-}$**: *UAS-mCD8-GFP, hsFlp122*; *MZ19-GAL4, UAS-mCD8-GFP/+*; *FRT82B, TubP-Gal80/FRT82B, Rab11*$^{EP3017}$ |
